# Molecular Insights into Red Palm Weevil Resistance Mechanisms of Coconut (*Cocos nucifera*) Leaves

**DOI:** 10.3390/plants13141928

**Published:** 2024-07-12

**Authors:** Li Liu, Wei Yan, Bo Liu, Weiquan Qin

**Affiliations:** Hainan Key Laboratory of Tropical Oil Crops Biology, Coconut Research Institute, Chinese Academy of Tropical Agricultural Sciences, Wenchang 571339, China; ywei123@catas.cn (W.Y.); liubo303@126.com (B.L.); qwq268@163.com (W.Q.)

**Keywords:** red palm weevil, *Cocos nucifera*, photosynthetic markers, metabolomics, transcriptomics

## Abstract

Red palm weevil (RPW) (*Rhynchophorus ferrugineus*) threatens most palm species worldwide. This study investigated the molecular responses of coconut (*Cocos nucifera*) leaves to RPW infestation through metabolomics and transcriptomics analysis. An RPW insect attack model was developed by placing different RPW larval densitiesin coconut plants and measuring the relative chlorophyll content of different leaf positions and physiological indicators of dysfunction after RPW infestation. The metabolomic changes were detected in the leaves of 10, 20, 30, 40, and 50 days after infestation (DAI) using GC-MS. Certain metabolites (glycine, D-pinitol, lauric acid, allylmalonic acid, D-glucaro-1, 4-lactone, protocatechuic acid, alpha, and alpha-trehalose) were found to be possible indicators for distinct stages of infestation using metabolomics analysis. The influence on ABC transporters, glutathione, galactose, and glycolipid metabolism was emphasized by pathway analysis. Differentially expressed genes (DEGs) were identified at 5, 10, 15, and 20 DAI through transcriptomics analysis of infested coconut leaves, with altered expression levels under RPW infestation. The KEGG pathway and GO analysis revealed enrichment in pathways related to metabolism, stress response, and plant–pathogen interactions, shedding light on the intricate mechanisms underlying coconut–RPW interactions. The identified genes may serve as potential markers for tracking RPW infestation progression and could inform strategies for pest control and management.

## 1. Introduction

The red palm weevil (*Rhynchophorus ferrugineus*) is the world’s most destructive insect pest for palm trees, frequently attacking both healthy and unhealthy specimens [1]. The larvae of the RPW feed inside the pical growth point of palm trees, causing severe damage to the tissues and compromising the structure of the palm stem. The principal hosts of RPWs are typically the most common landscape palms. According to several sources, approximately 132 insect and mite species are reported to attack date palm plants globally [2,3,4]. The RPW is the most significant stem and trunk borer of palm plants worldwide, with its larvae causing harm by feeding internally on the soft tissues of the trunk [5]. RPW engages in a short flight outside the tree as an adult to find a mate [6]. After mating, the female weevil lays eggs near the concealed petiole, or growth point. The hatched larvae then burrow into the tree trunk, causing damage for 2–3 months [7]. Early detection of these larvae is challenging due to their location within the tree trunk [8,9]. When symptoms are observable, most of the tree trunk is already hollowed out, leaving the tree unsalvageable [10]. Despite domestic and international research efforts [11,12] to prevent and control RPW, the rampant spread of the pest remains largely unaffected [13]. At least 15% of the world’s countries that cultivate coconuts and almost 50% of those that grow date palms are predicted to be at risk of RPW destruction, which might result in significant losses in palm oil production and related businesses [14,15]. RPW is regarded as an epidemic pest that attacks up to 40 kinds of palm trees, resulting in significant financial losses for plant owners worldwide [16]. 

The scientific community unanimously believes that early detection is essential for successfully preventing and controlling this pest [17,18]. Detecting insect-induced plant defense responses may assist in the early detection of RPW. Over time, plants have evolved to form induced defense responses under the stress of herbivorous insects. The early stages of this response involve changes in the transmembrane potential of plants and Ca^2+^ concentration within seconds or minutes of damage [18,19]. These changes usually occur when specific elicitors in the oral secretions of herbivorous insects participate in the regulation. The subsequent involvement of protein kinases in plant–insect interaction regulation is linked with relevant signaling pathways [20]. The activation of these pathways elevates the expression level of several defense genes and synthesizes related defense compounds [21]. Plants defend against pests through direct and indirect methods [22,23]. Direct defenses include chemical production like poisonous alkaloids and protease inhibitors, which deter or hinder insect feeding. Indirect defenses involve the release of plant volatile compounds, such as herbivore-induced plant volatiles (HIPVs), which attract natural enemies of pests for defense. This mechanism is crucial to the tri trophic interaction. HIPVs, which can attract natural enemies and serve as the communication medium between plants, are systemic. They can spread from the affected parts to the whole plant and even extend to surrounding healthy plants, thereby triggering the defense response of healthy plants and enhancing the insect resistance of plant populations [24].

By understanding the genetic basis of plant defense mechanisms against RPW, researchers can identify specific molecular markers associated with these responses. These markers can be used to develop diagnostic tools for the early detection of RPW infestations [25]. For instance, changes in the expression levels of certain genes or proteins in response to RPW attacks can serve as indicators of infestation [26,27,28]. In addition, knowledge of the genetic pathways involved in plant defense responses can aid in the development of biochemical sensors capable of detecting volatile organic compounds (VOCs) emitted by plants under stress due to RPW infestation. These sensors can detect subtle changes in the composition of VOCs, providing an early warning system for RPW presence before visible symptoms appear [29]. Furthermore, understanding the genetic basis of plant resistance to RPW can facilitate the breeding of resistant cultivars. By identifying genes responsible for conferring resistance, breeders can introgress these traits into commercially important palm species. This can reduce the susceptibility of palms to RPW attacks, thereby minimizing the need for intensive monitoring and control measures [30,31].

Research on the food preferences of RPW among different date palm varieties, including Mazafati, Rabbi, Halileh, Zardan, Pimazoo, and a native wild palm, *Nannorrhopsritchiana*, revealed varying levels of larval mortality, with Zardan showing the highest and Halileh the lowest. Wild palm exhibited complete pupal mortality. Analysis of vascular tissues and nutrient content indicated that sugar and calcium significantly influenced RPW’s vital parameters. Sugar was linked to growth and oviposition rates, while increased calcium inhibited RPW growth. The role of nutrient interactions in determining the food preferences of RPW and life cycle characteristics has been determined [32,33]. As a result of advancements in sequencing technology, genomic approaches to insect management have been proposed [34,35,36]. Initially, transcriptome analyses were conducted on RPW to pinpoint particular genes primarily active during its developmental stages and when infecting palm trees [37]. Moreover, through the genome assembly of RPW, the chromosomal-level resolution has been studied, which made it easy to identify crucial gene families associated with the destructive life history characteristics of RPW [38].

In several countries, particularly those in the Middle East, North Africa, and China, the production of coconuts (*C. nucifera*) is suffering due to the RPW herbivory. In China, it was first noted in Zhongshan City, Guangdong Province, in 1997. Since then, the presence of RPW has been reported in several provinces (Fujian, Hainan, Jiangsu, Yunnan, and Zhejiang), autonomous regions (Tibet and Guangxi), and the special administrative region (Hong Kong) of China. [39]. This invasion not only threatens the coconut industry but also poses ecological risks to China’s coastal areas. RPW is classified as highly harmful by Chinese agricultural and forestry authorities due to its significant impact on agriculture [40,41]. 

Several strategies and techniques exist for managing and preventing RPW attacks on coconut plants, but their effectiveness largely depends on early detection [8]. Currently, controlling the weevil involves the application of insecticides, the elimination of infected palm trees, and mass-trapping of adult weevils [42]. However, there is an issue with the overuse of pesticide treatments. This could potentially be addressed by developing more effective early detection approaches, which, when combined with innovative control strategies, would result in more long-lasting control [43]. As we know, the understanding of plant responses involves the activation of pathways producing chemicals to repel herbivores and the induction of the biosynthesis of various compounds, including plant growth regulators and metabolites involved in defense mechanisms. While insect responses entail increased biosynthesis of secondary metabolites, the knowledge of these pathways in RPW infected coconut plants is limited but essential for understanding coconut responses to RPW attacks [20]. 

A previous study provided a foundation for devising early and late RPW attack detection methods where the physiological and transcriptional responses of coconut leaves to RPW infestation over different time intervals (5, 10, 15, 20, and 25 DAI) were investigated. Higher populations of RPW insects corresponded with elevated antioxidant enzyme activities in coconut leaves. Additionally, transcriptomic analysis revealed significant differential expression of genes associated with plant–pathogen interactions, phenylpropanoid/flavonoid biosynthesis, amino sugar and nucleotide sugar metabolism, plant hormone signal transduction, mitogen-activated protein kinase, and reactive oxygen scavenging pathways. The findings of the study not only shed light on the molecular mechanisms underlying coconut–RPW interactions but also highlight potential candidate genes for manipulation in developing host–plant resistance strategies [44]. In this study, we aimed to bridge this gap by studying the metabolomics and transcriptomic responses of coconut cultivars to RPW infestation, using different infestation densities and time points for evaluation. The study used GC-MS-based metabolomic methods and transcriptomic analyses to explore the biochemical and genetic responses of other palm species (i.e., date palm and/or canary palm) to RPW infestation or other similar insect infestations. The research aims to identify potential diagnostic markers for the early prediction of RPW insect infection.

## 2. Results

### 2.1. Analysis of Photosynthetic-Related Markers of Coconut Leaf Samples after RPW Infestation

**Figure 1:**
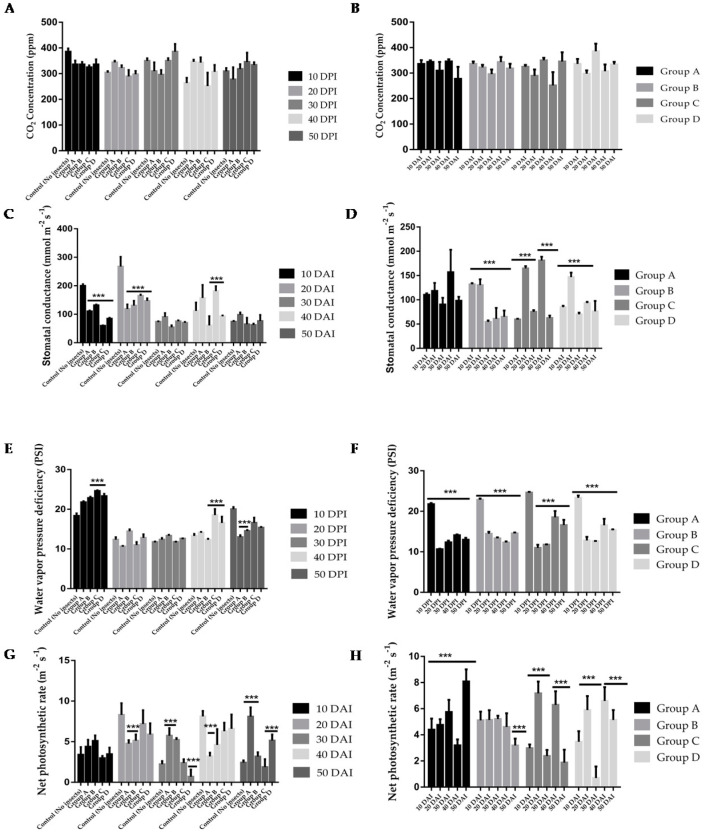

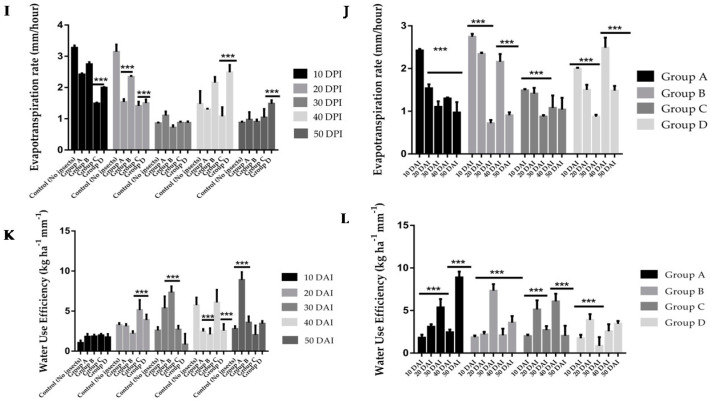
Status of photosynthetic-related markers of coconut leaf samples after RPW infestation. (**A**) CO_2_ concentration remained unchanged across all time points and groups. (**B**) CO_2_ concentration remained stable from 10 to 50 DAI. (**C**) Stomatal conductance showed varied responses, with significant increases observed in group C at 20 DAI and 40 DAI. (**D**) Stomatal conductance decreased significantly in later stages (30, 40, and 50 DAI) in group B and mostly decreased in group D, except for an increase at 20 DAI. Group C showed significant increases at 20 and 40 DAI but decreases at other time points. (**E**) Water vapor pressure deficiency did not show consistent trends indicative of infestation across the experimental period. (**F**) Water vapor pressure deficiency decreased significantly at later stages (20, 30, 40, and 50 DAI) compared to 10 DAI. (**G**) Net photosynthetic rate exhibited a significant decrease at 30 DAI in group D, despite decreases observed in group A at 30 and 50 DAI. (**H**) Net photosynthesis rate in group A significantly increased at 50 DAI, whereas groups B and C showed significant decreases. Group D exhibited a decrease at 30 DAI but increases at 20 and 40 DAI. (**I**) In groups C and D samples, a decreased evapotranspiration rate was observed at 20 DAI, whereas group D samples exhibited a significantly higher rate at 40 and 50 DAI. (**J**) Evapotranspiration rate decreased in later stages in Groups A and C but increased significantly in group D at 40 DAI. Group B showed higher rates at 10 DAI. (**K**) Water use efficiency significantly decreased at 40 DAI in groups A, B, and D, with group A showing a significant increase at 50 DAI. (**L**) Water use efficiency significantly increased at 50 DAI in group A, at 30 DAI in group B, at 40 DAI in group C, and at 20 DAI in group D. In groups C and D, water use efficiency significantly decreased at 30 DAI. Data were presented as mean ± SD and analyzed by one-way ANOVA, *** *p* < 0.001.

Detailed measurements of several key factors related to the cellular respiration of coconut plants in both control and infested groups were investigated. These parameters encompassed carbon dioxide (CO_2_) concentration, stomatal conductance, water vapor pressure deficiency, and the net photosynthetic rate. Throughout the timeline, the CO_2_ concentration in both control and infested groups was not significantly changed (Figure 1A). Additionally, water vapor pressure deficiency and the evapotranspiration rate also did not reveal a discernible trend that could serve as diagnostic markers (Figure 1E,I). Despite apparent differences in some groups, an overall trend was lacking in these two parameters.

Stomatal conductance measurements presented a more intriguing trend. Following a 20-day infestation period, an increase in stomatal conductance was noticeable in group A. However, this increase did not mirror the response in groups exposed to a higher quantity of RPW insects. At 40 DAI, stomatal conductance increased significantly in group C leaf samples. Groups A, B, C, and D samples at 10 and 20 DAI were noted to have significantly decreased stomatal conductance. While, at 30 DAI and 50 DAI, almost similar stomatal conductance was observed among all groups (Figure 1C). The reason for this differential response warrants further investigation. An unexpected yet intriguing discovery was the behavior of the net photosynthetic rate and water use efficiency. At 50 DAI, an increase was observed across all groups, with group A exhibiting the most significant candidate (Figure 1G,K). The net photosynthetic rate was significantly decreased at 20 DAI in group A and B samples, at 30 DAI in group D samples, and at 40 DAI in group A samples, while at 30 DAI in group A samples, it was significantly increased (Figure 1G). This counterintuitive finding, where infestation led to an increase in photosynthetic activity and water use efficiency, could indicate some form of compensatory or adaptive response of the plant to the stress of infestation. Although several factors traditionally associated with cellular respiration proved unhelpful in distinguishing between infested and non-infested plants, specific markers related to photosynthetic activity emerged as promising indicators of infestation. These markers could effectively identify the late stage of infestation when the quantity of RPW is relatively small.

Water vapor pressure deficiency was observed to increase significantly at 10 DAI and 40 DAI in group C leaf samples. But, at 50 DAI, water vapor pressure deficiency was decreased significantly in group A samples. Leaf samples from other groups showed almost similar values throughout the experimental period (Figure 1E).

The evapotranspiration rate decreased significantly in group A samples at 10 DAI and in group A, C, and D leaf samples at 20 DAI. But a significantly increased evapotranspiration rate was observed increased in group D leaf samples at later infestation stages (40 DAI and 50 DAI). Very little difference was observed in the evapotranspiration rate of leaf samples from other infestation groups throughout the experimental period (Figure 1E). Water use efficiency was observed to significantly increase in group C samples at 20 DAI, in group B samples at 30 DAI, and in group A samples at 50 DAI. An opposite trend was observed at 50 DAI in group A, group B, and group D leaf samples, respectively (Figure 1K). 

The concentration of CO_2_ remained relatively stable from 10 DAI to 50 DAI (Figure 1B). In group B, stomatal conductance exhibited a significant decrease during the later stages of infestation (30, 40, and 50 DAI). A similar trend was observed in group D samples, except at 20 DAI, where a significant increase was noted. Conversely, in group C samples, stomatal conductance increased significantly at 20 DAI and 40 DAI, while it decreased significantly at other time points (Figure 1D). The water vapor pressure deficit decreased notably during the later stages of infestation (20, 30, 40, and 50 DAI) compared to 10 DAI. In group A samples, the net photosynthesis rate significantly increased at 50 DAI, whereas in groups B and C, a significant decrease was observed at 50 DAI compared to earlier stages of infestation (Figure 1F). In group D samples, net photosynthesis decreased significantly at 30 DAI. Group C (20 DAI and 40 DAI) and D (20 DAI) samples showed significantly increased net photosynthesis levels (Figure 1H). In groups A and C samples, a decreased evapotranspiration rate was observed during the later stages of infestation, whereas group D samples exhibited a significantly higher rate at 40 DAI. Group B samples had a higher evapotranspiration rate during the earlier stage of infestation (10 DAI) (Figure 1J). Water use efficiency was significantly higher at 50 DAI in Group A samples, whereas in groups B, C, and D, higher efficiencies were observed at 30 DAI, 40 DAI, and 20 DAI, respectively. Interestingly, while significantly increased water use efficiency was noted at 30 DAI in groups A and B samples, it significantly decreased in groups C and D samples (Figure 1L).

### 2.2. Analysis of Chlorophyll Levels of Coconut Leaf Samples after RPW Infestation

**Figure 2:**
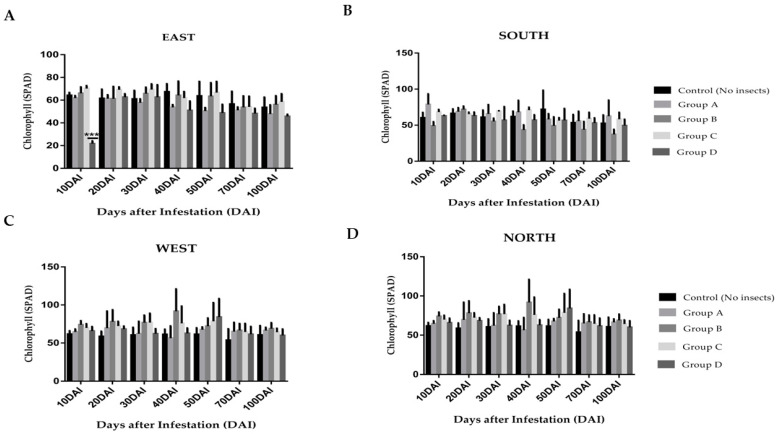
The chlorophyll levels in coconut leaf samples across different orientations (east, west, north, and south) and infestation levels. (**A**) Significant changes in chlorophyll levels were observed on the east side in group D at 10 DAI. (**C**) West-side-oriented leaves exhibited decreased chlorophyll levels in group A compared to the control group throughout the infestation period. (**B**,**D**) Subtle changes in chlorophyll levels were noted in south- and north-side-oriented leaves. Data were presented as mean ± SD and analyzed by one-way ANOVA.

In the broader scope of this investigation into the physiological responses of *C. nucifera* to RPW infestations, the alterations in chlorophyll levels in the leaves across four distinct orientations, east, west, north, and south, were investigated. The study design encompassed coconut leaves infested with varying RPW densities, enabling the comparison of multiple infestation levels. During the initial stages of a severe infestation (10 DAI), in group D, a significant change was observed in the chlorophyll levels of leaves on the east side of the plant. However, this alteration gradually diminished over the subsequent period from 20 to 70 days after infestation (Figure 2A). This distinctive early-stage change was specific to the heavy infestation group, with leaves in groups A, B, and C not exhibiting such noticeable differences in the eastward orientation.

Consequently, the east-side chlorophyll levels in these groups were not identified as reliable indicators of infestation. West-side-oriented leaves exhibited intriguing trends as well. Differences emerged between the control group (CK) and the lightly infested group A throughout the entire duration, from day 10 to day 100 post-infestation. The data suggest a progressive reduction in chlorophyll levels from the early to the late stages of infestation under light RPW attack (Figure 2C). When comparing the heavy infestation group D to the control group, west-side leaves also displayed a decrease in chlorophyll levels, though this change was not as pronounced as the one observed in the eastward leaves (Figure 2A,C).

Regarding the leaves on the south and north sides of the coconut palm, changes in chlorophyll levels were found to be rather subtle (Figure 2B,D). Therefore, these were not considered valuable indicators of infestation. The orientation of leaves, combined with the intensity of RPW infestation, appears to influence chlorophyll levels in coconut palms to varying degrees. These findings added another layer to our understanding of plant responses to pest infestations, although the specific mechanisms underlying these orientation-related changes remain unrevealed.

### 2.3. Analysis of Metabolomics Differences in Coconut Leaf Samples after RPW Infestation

**Figure 3:**
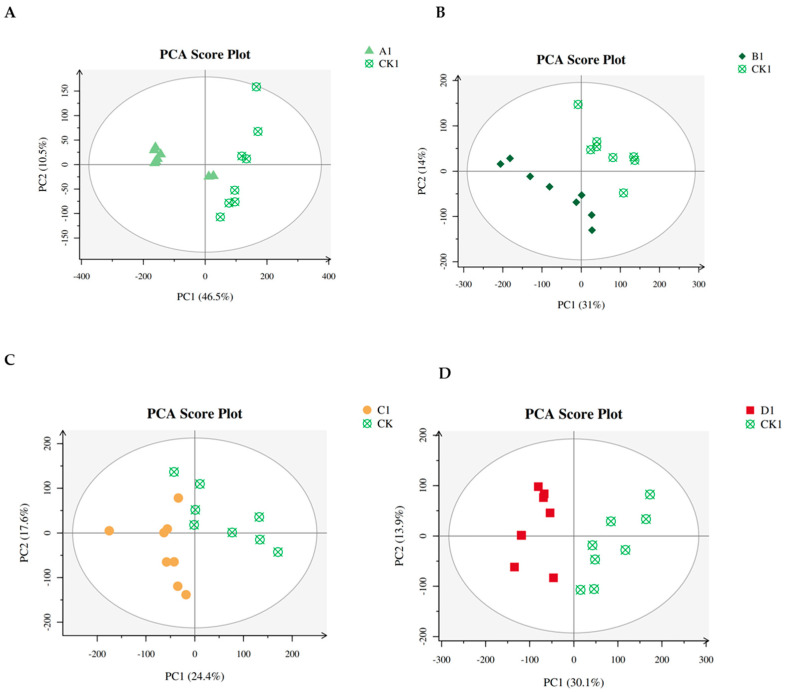
PCA score plots of infestation groups. Metabolomics analysis of coconut leaf samples post-RPW infestation revealed significant alterations in metabolite profiles compared to control samples. PCA plots (**A**–**D**) highlighted distinct metabolic shifts in infested groups (**A**–**D**) relative to controls, indicating a close association between RPW infestation and metabolic changes.

The PCA plot, contrasting group A (infested leaves) with the control group (non-infested leaves), unveiled an alteration in the metabolite profiles subsequent to infestation. This finding implies a close association between metabolic changes and RPW infestation (Figure 3A).

**Figure 4:**
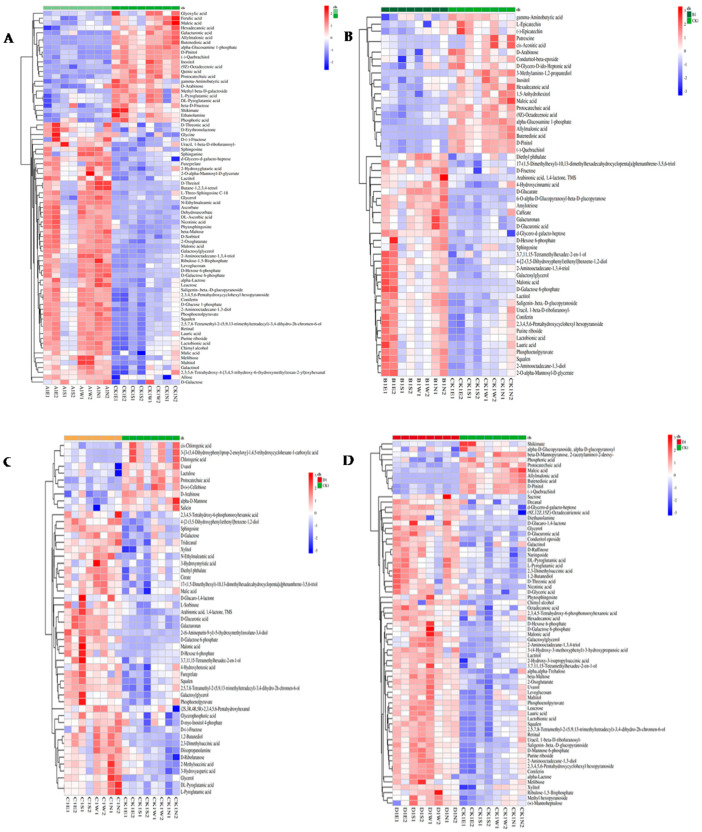
Differentially expressed metabolites in *C. nucifera* leaves. Heatmap showing metabolite profile changes after RPW infestation in group A (**A**), group B (**B**), group C (**C**), and group D (**D**) leaves.

**Figure 5:**
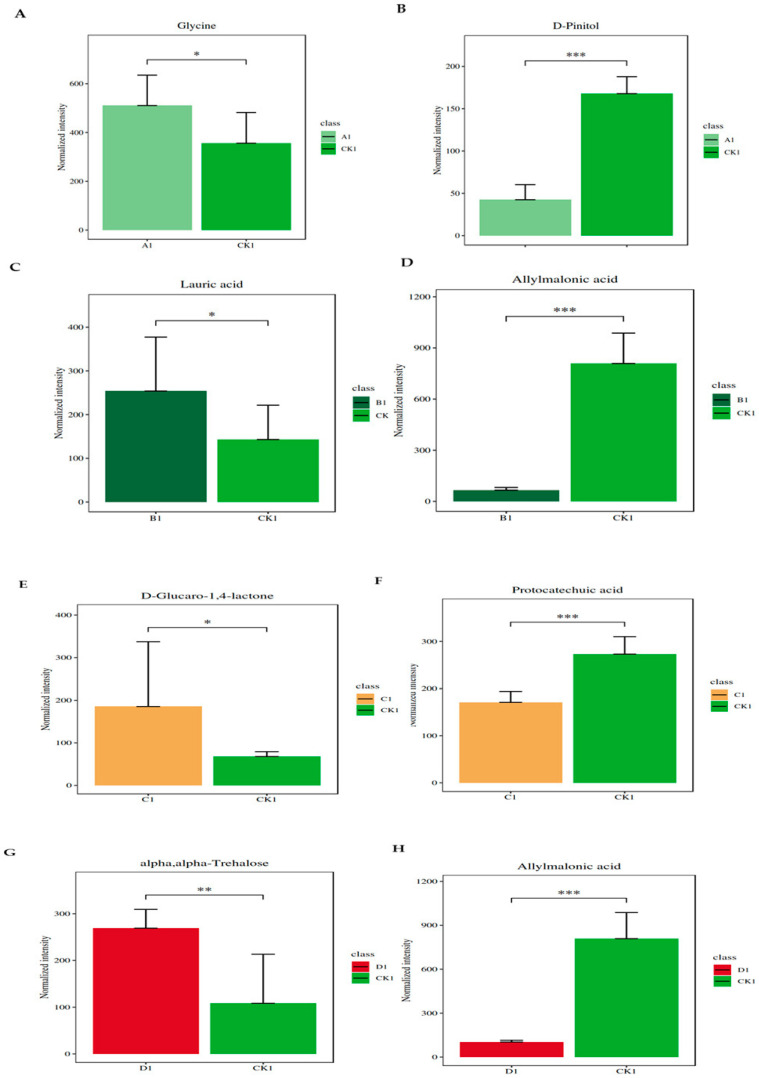
Differentially expressed metabolites in *C. nucifera* leaves. (**A**,**B**) The most up-regulated and down-regulated metabolite of group A. (**C**,**D**) The most up-regulated and down-regulated metabolite of group B. (**E**,**F**) The most up-regulated and down-regulated metabolite of group C. (**G**,**H**) The most up-regulated and down-regulated metabolite of group D (* *p* < 0.05, ** *p* < 0.01, *** *p* < 0.001).

To complement our analysis, a heatmap was utilized to visually represent changes in metabolite expression, effectively illustrating variability across different conditions (Figure 4A). The heatmap pinpointed glycine, a commonly found amino acid, as the most significantly increased metabolite post-infestation (Figure 5A). In contrast, D-pinitol, a widely distributed inositol ether in plants, emerged as the most substantially decreased metabolite following the RPW attack (Figure 5B). Given the significant shifts in the levels of these metabolites, both glycine and D-pinitol emerge as potential diagnostic markers for early-stage or light RPW infestation. These markers could enhance early detection, potentially enabling more timely and effective pest management strategies.

Table S1 provides a summary of results for various metabolic pathways. The path-ways are ranked based on their FDR values. According to the obtained results, galactose metabolism stands out as the most significant pathway with a very low FDR of 4.80 × 10^−7^, which is followed by glutathione metabolism, ABC transporter associated pathways, ascorbate and aldarate metabolism, and sphingolipid metabolism, respectively, with FDR values ranging from 0.030 to 0.160. Phenylalanine, tyrosine, and tryptophan bio-synthesis, glycerolipid metabolism, and butanoate metabolism pathways were identified as moderately significant pathways (0.05 < FDR < 0.1). Glutathione metabolism and ascorbate and aldarate metabolism have the highest impact scores, indicating potential biological relevance, and porphyrin and chlorophyll metabolism have the lowest impact score among all of the pathways. Comparative metabolomic analysis between group A and control samples highlighted the significance and potential impact of pathways such as galactose metabolism, glutathione metabolism, and ABC transporter processing, providing valuable insights into the molecular processes within the studied biological system.

**Figure 6:**
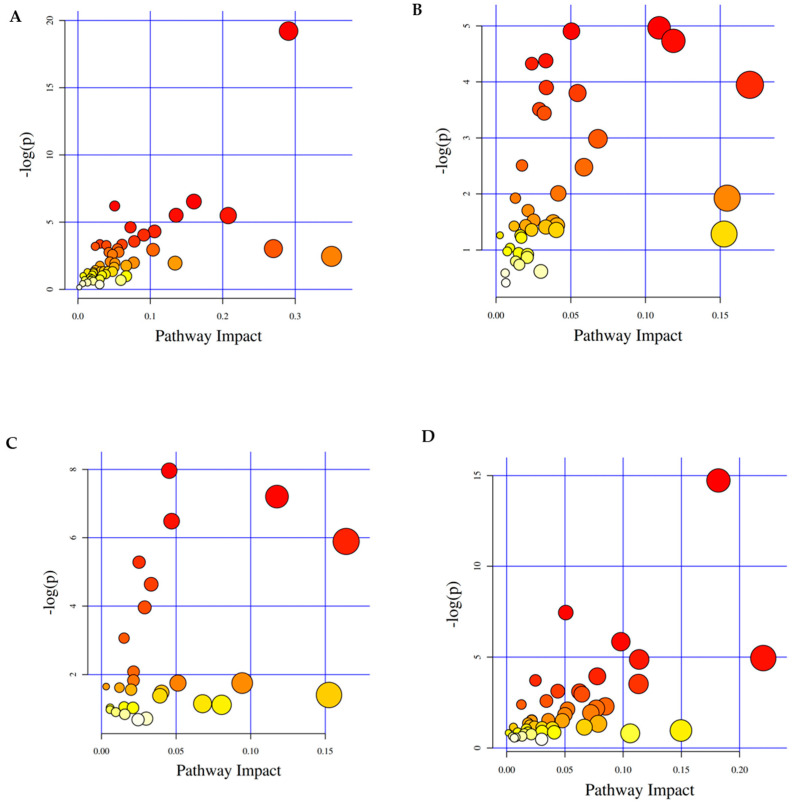
Graphical display of KEGG enrichment analysis of infestation groups A, B, C, and D samples. The degree of KEGG enrichment is measured by rich factor, q-value and the number of genes enriched in that pathway. The larger the rich factor, the greater the degree of enrichment. The smaller the q-value, the more significant the enrichment. About 20 of the most significantly enriched pathway entries were selected to display these figures (**A**–**D**).

To further explore the biochemical mechanisms behind these changes, we delved into the pathway differences between the infested and control groups (Figure 6A–D). This analysis underscored glutathione metabolism as the pathway most impacted by infestation. 

The PCA plot comparing group B and the control group revealed a substantial shift in metabolite profiles post-infestation, as illustrated in Figure 3B. Further insights into the variation of metabolite changes were depicted in a heatmap (Figure 4B), identifying the most significant metabolite changes after infestation. Lauric acid, a saturated fatty acid featuring a 12-carbon atom chain, emerged as the most significantly increased metabolite in the infested group (Figure 5C). Conversely, the most notable decrease was observed in allylmalonic acid, a crucial compound in plant metabolism (Figure 5D).

The noteworthy changes in the concentrations of these fatty acids suggest that both lauric acid and allylmalonic acid could potentially serve as diagnostic markers for medium RPW infestation. These findings open up new possibilities for early detection and proactive management of pest infestations in coconut plants. A deeper analysis of the biochemical processes underlying these changes involved exploring pathway differences between the infested and control groups (Figure 6B). 

Table S2 presents results for various metabolic pathways that were identified by comparing the metabolomics results between group B and control coconut leaf samples. The pathways were ordered by FDR values, providing insights into their statistical significance and potential biological relevance. Galactose metabolism, inositol phosphate metabolism, and ascorbate and aldarate metabolism were among the most significant pathways, with FDR values ranging from 0.050 to 0.280, while the citrate cycle (TCA cycle), fatty acid biosynthesis, sphingolipid metabolism, phenylpropanoid bio-synthesis, etc., were categorized as moderately significant pathways (0.05 < FDR < 0.1). Galactose metabolism, ascorbate and aldarate metabolism, and the citrate cycle (TCA cycle) have relatively high impact scores, suggesting potential biological importance.

The comparison of the PCA plot between group C and the control group highlighted a metabolite change after infestation (Figure 3C). Differentially changed metabolites are shown in a heatmap (Figure 5E). This analysis identified D-glucaro-1,4-lactone, a del-ta-lactone, as the most significantly increased metabolite post-infestation (Figure 5E). Conversely, protocatechuic acid, a phenolic acid and a major metabolite of antioxidant polyphenols in plants, exhibited the most significant decrease (Figure 5F). 

Various metabolic pathways were identified through analysis of the metabolomics data between group C and control coconut leaf samples (Table S3). Pentose and glucuronate interconversions, glycerolipid metabolism, galactose metabolism, and glycolysis/gluconeogenesis stand out as highly significant pathways, with FDR values ranging from 0.025 to 0.073. Other pathways including ABC transporter-mediated pathways, inositol phosphate metabolism, and carbon fixation in photosynthetic organisms were classified as moderately significant pathways.

The metabolites identified from this analysis could be used as potential diagnostic markers for medium RPW infestations. Monitoring the levels of these chemical substances in coconut plants could allow for the early detection of infestations, facilitating more effective control measures. To comprehend the mechanisms driving these metabolite changes, the differences in biochemical pathways between the infested and control groups were examined (Figure 3D). These identified metabolites have the potential to be used as diagnostic markers for medium-sized RPW infestations because of their striking variations in presence. We may be able to identify infestations early on and take more effective management action by monitoring these chemical compounds in coconut plants. Variations in metabolic pathways between the infested and control groups were investigated in order to comprehend the processes behind these metabolite changes. We discovered that the glycerolipidmetabolism was the pathway most affected by RPW infection. The production of glycerolipids such as monoacylglycerols (MAGs) and diacylglycerols (DAGs), which are necessary for the construction of membranes, the storage of calories, and a number of critical intracellular signaling activities, is supervised by this route.

Changes in the metabolite profiles following infestation were shown by the PCA plot that contrasted group D with the control group (Figure 6D). This significant change in metabolite dynamics highlights the significant effect that RPW infection has on the metabolic systems of coconut plants. To identify metabolite changes, a heatmap was used (Figure 4D), which showed differences in metabolite changes. The metabolite with the most rise among the metabolites was alpha-trehalose, a disaccharide made up of two glucose molecules (Figure 5G). On the other hand, allylmalonic acid, a metabolite essential to plant physiology, showed a significant decline (Figure 5H). The measurement of the levels of alpha-trehalose and allylmalonic acid in coconut plants could serve as potential diagnostic markers for RPW infestation. This early detection holds promise for facilitating timely intervention and enhancing pest management. Further exploration of the distinctions between heavily infested and control groups identified ABC transporters as the most impacted markers (Figure 6D). 

ABC transporter-mediated pathways, pentose and glucuronate interconversions, starch and sucrose metabolism, glycerolipid metabolism, and the citrate cycle (TCA cycle) were detected as highly significant pathways in comparison to the metabolomics analysis of group D and control coconut leaf samples (Table S4). Glycolysis/gluconeogenesis, the bio-synthesis of unsaturated fatty acids, and phenylalanine, tyrosine, and tryptophan bio-synthesis were also some other significant pathways. From the analysis of group D samples, it was observed that ABC transporter-mediated pathways, pentose and glucuronate interconversions, and starch and sucrose metabolism have potential biological importance. ABC transporters play pivotal roles in various biological processes in plants, encompassing pathogen response, surface lipid deposition, seed phytate accumulation, and the transport of phytohormones, such as auxin and abscisic acid (ABA). Consequently, monitoring the activity of ABC transporters and their associated biological processes offers an additional avenue for detecting and assessing the severity of RPW infestations.

This research investigated metabolite profiles across different infestation levels in *C. nucifera* leaves. Significant changes were observed in metabolite profiles at different infestation severities, identifying distinct metabolites, such as glycine, D-pinitol, lauric acid, allylmalonic acid, D-glucaro-1,4-lactone, proto-catechuic acid, alpha, and alpha-trehalose, as potential markers. We also found that infestation impacts various metabolic pathways, including glutathione, galactose, and glycerolipid metabolism, as well as the activity of ABC transporters. These findings could provide crucial early diagnostic tools for pest management and highlight the significant metabolic impact of RPW infestations on coconut plants.

### 2.4. Transcriptome Analysis of Artificially Infected Coconut Leaves

In this study, transcriptome sequencing of about 75 samples was carried out, and a total of 533.59 Gb of clean data were generated. The clean data of each sample reached up to 6 Gb, and the Q30 base percentage was ≥93%. Differentially expressed genes (DEGs) were identified based on their expression levels in different samples and performed functional annotation and enrichment analysis on them. Based on the alignment results and the gene’s position information on the reference genome, the number of reads for each gene was counted. The number of fragments in a transcript is related to the amount of sequencing data (or mapped data), transcript length, and transcript expression level. In order for the number of fragments to truly reflect the transcript expression level, it is necessary to compare the number of mapped reads in the sample and the transcript expression level. FPKM was used as an indicator to measure transcript or gene expression levels. Typically, the expression levels of protein-coding genes that can be sequenced have FPKM values spanning 10^−2^ to 10^4^. 

### 2.5. Identification of DEGs between the RPW Infestation Conditions

The significance of DEGs under RPW infestation conditions was assessed using the criteria of a false discovery rate (FDR) less than 0.05 and an absolute log_2_ ratio greater than 2. Table S5 represents the total number of DEGs, the number of up-regulated genes, and the number of down-regulated genes in each group. The highest number of differentially expressed genes (5373) were identified in the 0503CK_vs._0503C groups, where 2632 genes and 2741 genes were down-regulated and up-regulated, respectively. A higher number of DEGs were identified in the following groups: 0428CK_vs._0503CK (5347 genes), 0428C_vs._0503C (5346 genes), 0428B_vs._0503B (5070 genes), 0428CK_vs._0518CK (5057 genes), 0428A_vs._0503A (4347 genes), 0428D_vs._0503D (4073 genes), etc. On the other hand, the lowest number of DEGs was identified in 0428B_vs._0513B (353 genes), where 230 genes and 123 genes were down-regulated and up-regulated, respectively. Other groups where the lower number of DEGs were observed were 0428B_vs._0508B (603 genes), 0503A_vs._0518A (638 genes), 0428C_vs._0513C (932 genes), 0503C_vs._0518C (941 genes), etc. It was noticed that a greater quantity of genes was activated when RPW attacked under B, C, and D densities compared to the control group.

Principal component analysis (PCA) and Pearson correlation were performed on the fragments per kilobase of transcript per million fragments mapped (FPKM) values. The Pearson correlation coefficient (R^2^) between samples exceeded 0.8, and clear clustering was observed, affirming the reliability of the transcriptome data for further analysis. Pearson’s Correlation Coefficient r (Pearson’s correlation coefficient) is used as an evaluation index for biological repeat correlation. The closer the absolute value of r is to 1, the stronger the correlation between the two repeated samples. The differential genes of all comparison groups were combined and used as a differential gene set for hierarchical clustering analysis. In the hierarchical clustering, similar expression changes have been observed in different sample groups. Genes have similar change trends under different experimental treatments and may have similar functions.

### 2.6. Genes Implicated in the Development of the RPW Attack in Coconut Leaves

Several genes were identified in the four treatment comparisons at different time points. Among these, in the 0428A_vs._0428B comparison group, *E3 ubiquitin-protein ligase UNKL (COCNU_16G006610)* had the highest FPKM values in A population density at five DAI. Other genes, including *glycerol-3-phosphate transporter (COCNU_07G005810)*, *chitinase (COCNU_scaffold006534G000030)*, *calmodulin (COCNU_05G007540)*, and *EREBP-like factor (COCNU_03G006840)*, showed higher FPKM values. All of these genes were down-regulated at 5 DAI. In the B population density group, genes including *hydroxyjasmonate sulfotransferase (COCNU_scaffold051069G000020)*, *E3 ubiquitin-protein ligase UNKL (COCNU_16G006610)*, *COCNU_07G005810 (glycerol-3-phosphate transporter)*, *annexin D (COCNU_16G002250)*, and *EREBP-like factor (COCNU_03G006840)* were identified with higher FPKM values. In the 0428A_vs._0428C comparison group, *E3 ubiquitin-protein ligase UNKL(COCNU_16G006610)*, *solute carrier family 15 (COCNU_01G009750)*, *shikimate O-hydroxycinnamoyltransferase (COCNU_01G004790)*, *oxalate---CoA ligase (COC-NU_09G007640)*, and *interleukin-1 receptor-associated kinase 4 (COCNU_scaffold008462G000030)* showed higher FPKM values. In the 0428 A vs. 0428 D comparison groups, genes including *ferric-chelate reductase (COCNU_scaffold009298G000030)*, *N-acylneuraminate-9-phosphatase (COCNU_16G002140)*, *COCNU_12G007620 (heat shock 70kDa protein)*, *MYB-related transcription factor LHY (COCNU_09G003420)*, *zinc finger protein CONSTANS (COC-NU_01G005950)*, and *L-galactose phosphorylase (COCNU_02G011760)* showed higher FPKM values in the A population density group. In contrast, genes including *HSP20 family protein (COCNU_02G002340)*, *carbonic anhydrase (COCNU_14G004980)*, *thioredoxin 1 (COC-NU_11G002270)*, *histone H1/5 (COCNU_03G002150)*, *COCNU_contig69145767G000010 (thioredoxin 1)*, and *phenylcoumaran benzylic ether reductase (COCNU_13G000790)* were identified with higher FPKM values in the D population group. 

At 5 DAI, the 0428 CK vs. 0428 A comparison group identified several genes including ferric-chelate reductase (COCNU_scaffold009298G000030), adenylyl-sulfate reductase (glutathione) (COCNU_06G011940), thioredoxin 1 (COC-NU_02G011730), and N-acylneuraminate-9-phosphatase (COCNU_16G002140), while genes including photosystem I subunit X (COCNU_scaffold049646G000020) and ferric-chelate reductase (COCNU_scaffold009298G000030) were identified in 0428 CK vs. 0428 B groups with higher FPKM values. In the 0428 CK vs. 0428 C group, genes such as adenylyl-sulfate reductase (glutathione) (COCNU_06G011940), lipoxygenase (COCNU_01G007010), and ferric-chelate reductase (COCNU_scaffold009298G000030) were identified. Higher expressions of genes including urease accessory protein (COCNU_06G014700), oxidoreductase (COC-NU_14G011580), coatomer subunit beta (COCNU_14G008950), and lutamine synthetase (COC-NU_03G002950) were identified in the 0428 CK vs. 0428 D groups.

At 10 DAI, in the 503 CK vs. 503 A group, isoprene synthase (COC-NU_scaffold029432G000010), granule-bound starch synthase (COCNU_08G005950), potassium channel (COCNU_01G020060), nitrate reductase (COCNU_scaffold002757G000010), coatomer subunit beta (COCNU_14G008950), and phenylcoumaran benzylic ether reductase (COCNU_13G000790) were identified with higher expressions.In the 503 CK vs. 503 B group, genes including nitrate reductase (COCNU_scaffold002757G000010), HSP20 family protein (COCNU_02G002340), ferredoxin-nitrite reductase (COCNU_scaffold000819G000030), coatomer subunit beta (COCNU_14G008950), phenylcoumaran benzylic ether reductase (COC-NU_13G000790), and isoprene synthase (COCNU_scaffold029432G000010); in the 503 CK vs. 503 C group, genes including phytepsin (COCNU_03G010620), phenylcoumaran benzylic ether reductase (COCNU_13G000790), flavin-binding kelch repeat F-box protein 1 (COC-NU_03G009890), coatomer subunit beta (COCNU_14G008950), phenylcoumaran benzylic ether reductase (COCNU_13G000790), and isoprene synthase (COCNU_scaffold029432G000010); and in the 503 CK vs. 503D group, lipoxygenase (COCNU_01G007010), fanconi anemia group D2 protein (COCNU_15G001970), urease accessory protein (COCNU_06G014700), chitinase (COCNU_scaffold006534G000030), phenylcoumaran benzylic ether reductase (COC-NU_13G000790), lipoxygenase (COCNU_01G007010), coatomer subunit beta (COC-NU_14G008950), urease accessory protein (COCNU_06G014700), andphenylcoumaran benzylic ether reductase (COCNU_13G000790) were identified as highly expressed genes.

At 15 DAI, in the 508 CK vs. 508 A group, genes such as heat shock transcription factor, other eukaryote (COCNU_03G001480), urease accessory protein (COCNU_06G014700), coatomer subunit beta (COCNU_14G008950), expansin (COCNU_02G007710), glutamate receptor, ionotropic, and plant (COCNU_contig69499688G000010); in the 508 CK vs. 508 B group, heat shock transcription factor, other eukaryote (COCNU_03G001480), HSP20 family protein (COCNU_02G002340), glutamate receptor, ionotropic, plant (COC-NU_contig69499688G000010), N-acylneuraminate-9-phosphatase (COCNU_16G002140), MYB-related transcription factor LHY (COCNU_09G003420), ornithine carbamoyl transferase (COCNU_09G008820), in 508 CK vs. 508 C group, oxidoreductase (COCNU_14G011580), urease accessory protein (COCNU_06G014700), glutamate receptor, ionotropic, plant (COC-NU_contig69499688G000010), and HSP20 family protein (COCNU_02G006340); and in the 508 CK vs. 508 D group, urease accessory protein (COCNU_06G014700), and coatomer subunit beta (COC-NU_14G008950) were detected with higher FPKM values. Many of those identified were not detected in the control groups.

At 20 DAI, genes including carbonic anhydrase (COCNU_14G004980), histone H1/5 (COCNU_08G006060), charged multivesicular body protein 5 (COCNU_15G005710), urease accessory protein (COCNU_06G014700), COCNU_06G011940 (adenylyl-sulfate reductase (glutathione), long-chain fatty acid omega-monooxygenase (COCNU_09G000070), adenylyl-sulfate reductase (glutathione) (COCNU_06G011940), HSP20 family protein (COC-NU_scaffold007600G000070), glutamate receptor, ionotropic, plant (COC-NU_contig69499688G000010), ferric-chelate reductase (COCNU_scaffold009298G000030), ferric reduction oxidase 7 (COCNU_09G003420), N-acylneuraminate-9-phosphatase (COC-NU_16G002140), photosystem I subunit X (COCNU_scaffold049646G000020), L-ascorbate peroxidase (COCNU_09G004370), GDP-L-galactose phosphorylase (COCNU_02G011760), (COCNU_14G008950), histone-binding protein RBBP4 (COCNU_06G008910), phenyl-coumaran benzylic ether reductase (COCNU_13G000790), and glutathione S-transferase (COC-NU_01G002110) were identified in 518 CK vs. 518 A, 518 CK vs. 518 B, 518 CK vs. 518 C, and 518 CK vs. 518 D comparison groups, respectively.

The comparisons between same infestation groups (A, B, C, and D) at different time intervals such as 5 DAI, 10 DAI, 15 DAI, and 20 DAI identified genes including *copper chaperone (COCNU_07G010820)*, *fructose-bisphosphate aldolase*, *class I (COC-NU_07G006240)*, *sulfate transporter 1*, *high-affinity (COCNU_13G002790)*, *heat shock 70kDa protein 1/2/6/8 (COCNU_12G007620)*, *charged multivesicular body protein 5 (COC-NU_15G005710)*, *glutathione S-transferase (COCNU_05G007300)*, *aquaporin TIP (COC-NU_07G009710)*, *premnaspirodiene oxygenase (COCNU_13G008330)*, *light-harvesting com-plex II chlorophyll a/b binding protein 5 (COCNU_scaffold007252G000060)*, *coatomer subunit beta (COCNU_14G008950)*, *adenylyl-sulfate reductase (glutathione) (COCNU_06G011940)*, *ferric-chelate reductase (COCNU_scaffold009298G000030)*, *E3 ubiquitin-protein ligase UNKL (COCNU_16G006610)*, *expansin (COCNU_scaffold034232G000010)*, *phenylcoumaran benzylic ether reductase (COCNU_13G000790)*, *urease accessory protein (COCNU_06G014700)*, *expansin (COCNU_02G007710)*, *pathogenesis-related protein 1 (COC-NU_scaffold034232G000010)*, *aspartyl protease family protein (COCNU_01G012730)*, and *dual specificity kinase (COCNU_05G004480)*. Most of the genes were identified and expressed at the later infestation stages, and a decreased expression trend was observed compared to the early infestation stages. The genes that were identified could serve as possible markers for tracking the progression of RPW attacks.

### 2.7. KEGG Pathway Analysis of Identified DEGs

**Figure 7:**
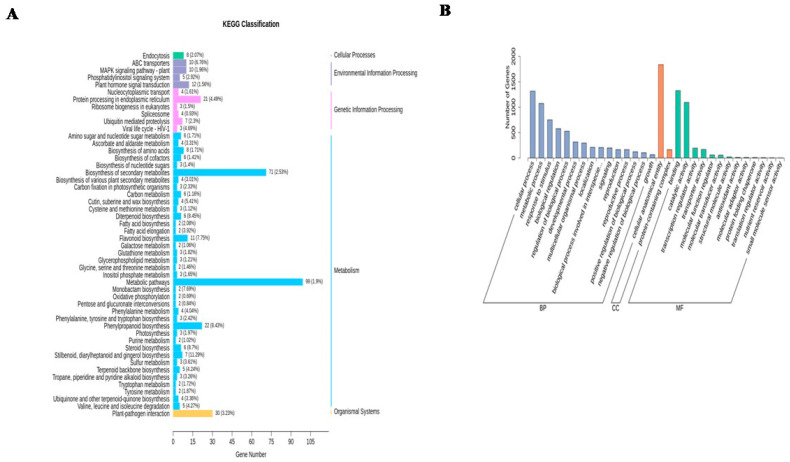
(**A**) KEGG pathways of DEGs. (**B**) GO classifications of DEGs.

The KEGG pathway chart is presented in Figure 7A. From the pathway analysis, it was observed that DEGs between different comparison groups showed significant enrichment in various pathways associated with metabolism, the organismal system, genetic information processing, environmental information processing, and cellular processes. These pathways encompass metabolic pathways, biosynthesis of secondary metabolites, plant–pathogen interaction, sucrose metabolism, the mitogen-activated protein kinase (MAPK) signaling pathway plant, protein processing in the endoplasmic reticulum, and endocytosis.

### 2.8. GO Analysis of Genes

Among the three GO classifications of biological process (BP), cellular component (CC), and molecular function (MF), there were 15, 2, and 13 functional groups, respectively. Within these groups, the terms cellular process (GO:0009987), cellular anatomical entity (GO:0110165), and binding (GO:0005488) emerged as dominant in each respective category. Additionally, the presence of genes was observed in functional groups such as metabolic process (GO:0008152), response to stimulus (GO:0050896), biological regulation (GO:0065007), regulation of biological process (GO:0050789), developmental process (GO:0032502), signaling (GO:0023052), protein-containing complex (GO:0065003), transporter activity (GO:0005215), and transcription regulator activity (GO:0140110). The statistical results of the GO classification of differentially expressed genes are shown in Figure 7B. 

## 3. Discussion

**Figure 8:**
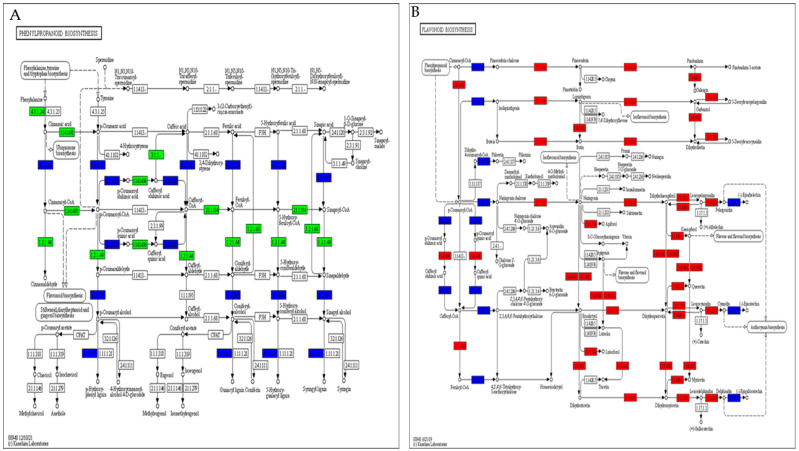
Significantly enriched pathways of the identified DEGs through transcriptomics analysis. (**A**) Phenylpropanoid biosynthesis pathway. (**B**) Flavonoid biosynthesis pathway.

In the current study, significant changes in chlorophyll levels were observed only in the heavily infested group D at the early stage (10 days post-infestation), which diminished over time in the east side leaves. In west side leaves, reductions in chlorophyll levels were observed from early to late stages, in both the control and lightly infested group A. The changes were less pronounced in the heavily infested group D. In the north and south leaves, changes in chlorophyll levels were subtle and not considered reliable indicators of infestation. In the current study, significant alterations in metabolite profiles were identified post-infestation, among which glycine levels increased and D-pinitol levels decreased in group A; lauric acid levels increased and allylmalonic acid levels decreased in group B; D-glucaro-1,4-lactone levels increased and protocatechuic acid levels decreased in group C; and alpha-trehalose levels increased and allylmalonic acid levels decreased in group D. Through transcriptomics analysis, specific genes such as *E3 ubiquitin-protein ligase*, *glycerol-3-phosphate transporter*, *chitinase*, and *calmodulin* showed differential expression under RPW infestation. DEGs showed significant enrichment in pathways related to metabolism, genetic information processing, and cellular processes (Figure 8A,B). Our previous study investigated the leaf physiological indicators, including activities of catalase (CAT), peroxidase (POD), superoxide dismutase (SOD), and malondialdehyde (MDA) content, in response to different RPW population densities and days after infestation (DAI). Variations in CAT, POD, and SOD activities and MDA content in response to RPW infestation were reported highlighting significant changes in CAT activity, with the highest decrease observed in RPW population density C. Several disease resistance proteins and EIX receptor genes showed higher expressions in coconut leaves infested with RPW, particularly in the C density, compared to the control (CK). Their absence or near-zero expression in the D density might contribute to the higher damage observed in those leaves. Genes associated with phytohormones such as abscisic acid (ABA), auxin, ethylene (ET), jasmonic acid (JA), salicylic acid (SA), and cytokinins showed differential expressions upon RPW attack. For instance, ABA and auxin genes exhibited either repression or enhancement in response to RPW, while SA-related genes were mostly induced upon RPW attack. JA-related genes, including *JA-amino synthetase*, were consistently expressed higher upon RPW attack. Furthermore, MAPK cascade-related genes, including *mitogen-activated protein kinase kinase (MAPKK)* and *LRR receptor-like serine/threonine-protein kinase FLS2* genes, showed differential expressions in coconut leaves under RPW attack. Additionally, genes involved in amino sugar and nucleotide sugar metabolism displayed varied expressions, with several chitinase and other metabolism-related genes expressed higher in the C density compared to the D density. Transcription factor families such as AP2/ERF, bHLH, MYB, and WRKY exhibited contrasting expression trends in response to the RPW attack, suggesting their potential roles in regulating the coconut leaf response to the infestation [44].

The carbon dioxide (CO_2_) status of palm trees can be significantly affected by RPW infestations. This observation starkly contrasts our initial expectations that infestation would not lead to a significant change in CO_2_ concentration. RPW infestations can lead to several changes in the CO_2_ status of palms due to the damage inflicted by the weevils on the plant’s vascular system and the disruption of photosynthesis. Decreased photosynthetic activity can lead to a decrease in CO_2_ uptake from the atmosphere [45]. As palm trees respond to RPW infestations and damage by initiating defense mechanisms, such as wound healing and the production of defensive compounds, there is an increase in metabolic activity, including respiration. This elevated respiration rate may result in higher CO_2_ released into the atmosphere [46]. RPW feeding leads to the wilting and eventual death of palm fronds. Wilted fronds are less effective in photosynthesis and gas exchange, disrupting the plant’s normal balance of CO_2_ [47]. RPW larvae burrow into the heart of palm trees, damaging vascular tissues. This damage can disrupt water and nutrient transport within the plant, indirectly impacting the plant’s ability to take up CO_2_ for photosynthesis [48]. RPW infestations can significantly influence the stomatal conductance status in palm trees. RPW infestations often induce stress in palm trees as a response to the damage inflicted by the weevils. This stress can lead to the closure of stomata to reduce water loss through transpiration, thereby decreasing stomatal conductance [49]. RPW infestations can disrupt the hydraulic balance in palm trees by damaging the vascular system. As a result, the water supply to the leaves may become compromised, leading to stomatal closure as a protective response to avoid excessive water loss. Stomatal conductance is closely linked to carbon assimilation through photosynthesis. Reduced stomatal conductance can result in decreased CO_2_ uptake, ultimately affecting the plant’s ability to produce sugars and support its growth and maintenance [50]. Prolonged closure of stomata and reduced stomatal conductance can lead to a decline in palm tree health, making them more susceptible to other stress factors and diseases [51]. The water vapor pressure deficiency (VPD) status in palm trees can be significantly affected by RPW infestations which can induce stress in palm trees, leading to stomatal closure and reduced transpiration rates. This reduction in transpiration limits the loss of water vapor from the leaf to the atmosphere, causing an increase in VPD [49]. RPW damage to the palm’s vascular system can disrupt hydraulic conductivity, leading to an imbalance in water uptake and distribution within the plant. This disruption can result in altered VPD [47]. The differences in photosynthetic-related factors were observed after infestation. The main physiological traits impacted by RPW infection were stomatal conductance, net photosynthetic rate, and water use efficiency. When examined collectively, these variables may be trustworthy indicators of a prolonged RPW infection of coconut trees. Regardless of the infestation’s prevalence, a noteworthy discovery was the variation in chlorophyll levels seen in the leaves of the coconut plants on their east and west faces. A plausible explanation for the subtler changes in chlorophyll levels in leaves on the north and south sides might be sunlight exposure. East-ward and west-ward-facing leaves are more likely to receive a higher intensity of sunlight, which could influence their chlorophyll content in the event of an infestation. This result is consistent with expectations because biotic stressors cause metabolic changes in plants that move from productivity (photosynthesis, growth, etc.) to survival (antioxidant generation, plant hormone production, etc.) [52,53]. Therefore, it is probable that the down-regulation of photosynthesis-related genes and proteins led to decreased photosynthetic activity in the infested *E. guineensis*. Additionally, the relationship between the host plant’s photosynthesis activity and the degree of RPW infestation differed from that found in a prior study that examined the photosynthesis activity of P. dactylifera palms infected with RPW. The effect of RPW infestation on the host’s physiology has been investigated in several studies. The ability of insect herbivory to alter temperature, growth, photo-synthetic activity, fruit output and reproductive potential has been demonstrated [54,55]. However, the kind of host and the invasive insects determine the physiological effects on the host plant. The herbivorous behavior of stem-borer insects does not affect the host plants consistently. A prior study on the impact of RPW infestation on *P. canariensis* discovered that the stomatal conductance of the infested palm was noticeably reduced [56]. A survey of *P. dactlyifera* identified that RPW infestation did not affect the stomatal conductance [57]. Therefore, it is suggested that different species have different physiological responses after RPW infestation. 

In metabolite analysis, glycine, D-pinitol, lauric acid, allylmalonic acid, D-glucaro-1,4-lactone, proto-catechuic acid, alpha, and alpha-trehalose are metabolites that emerged as potential markers for the corresponding stages of infestation in this study. D-pinitol, also known as 3-O-methyl-chiro-inositol, is a cyclic sugar alcohol found in various plants and is considered a compatible solute, contributing to osmotic regulation and stress tolerance [58]. Some studies suggest that D-pinitol may exhibit antioxidant properties [59]. Protocatechuic acid is a phenolic compound found in various plants and is known for its antioxidant properties. While the specific role of protocatechuic acid in plant defense mechanisms may vary, its antioxidant activity suggests a potential role in protecting plants from oxidative stress caused by ROS [60]. Phenolic compounds, including protocatechuic acid, are part of the plant’s secondary metabolites, may inhibit the growth of pathogens, and contribute to the overall resistance of the plant [61]. Lauric acid is a fatty acid with known antimicrobial properties, and these properties could potentially contribute to plant defense against microbial pathogens [62]. Allelopathy involves the release of biochemical compounds by one plant species that can influence the growth, development, and survival of other nearby plants [63]. Alpha, alpha-trehalose, commonly referred to as trehalose, is a disaccharide sugar studied for its potential role in plant stress responses. Trehalose is involved in various physiological processes, including sugar signaling, osmoregulation, and protection against environmental stresses [64]. Trehalose has been implicated in protecting plants from oxidative stress caused by ROS. ROS are generated under various stress conditions, and trehalose may help scavenge these radicals, preventing damage to cellular structures [65], and trehalose can regulate stress-responsive genes [66].

The metabolite analysis of group A- and B-infested leaves identified galactose metabolism as the most significant pathway (also in group C as one of the most significant pathways). Galactose metabolism plays a crucial role in plant defense responses against biotic stress. The galactose metabolism pathway emerged as the most impacted, and given its tight regulation in plants, measuring galactose or its breakdown product, glucose, could offer another method for detecting RPW infestation. The interplay of galactose and its derivatives is intricately linked to the modulation of various defense mechanisms in plants. For example, galactose and its derivatives are involved in the biosynthesis of cell wall components, which are integral to the reinforcement of plant cell walls and act as physical barriers against pathogen invasion [67,68]. Additionally, galactose-containing compounds, such as galactolipids, are essential constituents of plant cell membranes and have been implicated in signaling pathways associated with plant defense responses [69,70]. Furthermore, the galactose metabolism pathway intersects with other metabolic pathways, contributing to the production of secondary metabolites with antimicrobial properties. For instance, galactose-derived precursors may participate in the synthesis of secondary metabolites like flavonoids and phytoalexins, which are known to have antimicrobial and antioxidant activities [71,72]. The regulation of galactose metabolism is dynamic and responsive to biotic stress signals. Pathogen attacks or other biotic stressors can trigger changes in the expression of genes related to galactose metabolism, influencing the production of defensive compounds and altering the overall metabolic landscape of the plant [73,74]. 

In group C samples, the pentose and glucuronate interconversion pathway is identified as the most significant pathway that is involved in various cellular processes, including the synthesis of nucleotides and the generation of precursors for cell wall components. While there may not be direct evidence linking this pathway to plant defense against biotic stress, its products and intermediates could contribute to the overall metabolic adjustments during stress responses [75]. The alterations in glycerolipid composition are often associated with stress responses. Changes in lipid profiles can affect membrane fluidity and the production of lipid-derived signaling molecules involved in defense signaling pathways [76,77]. 

Another metabolic pathway related to ATP-binding cassette (ABC) transporters was identified as a significant pathway in group C leaf samples. ABC plays a role in transporting secondary metabolites, phytohormones, and defense-related compounds. They contribute to the plant’s defense by regulating the intracellular concentration of these molecules [78]. ABC transporters play a crucial role in the plant defense response against biotic stress. These transporters are integral components of the cellular machinery responsible for transporting various molecules, including secondary metabolites, across cellular membranes. In the context of plant defense, ABC transporters are involved in the transport of defense-related compounds, thereby contributing to the plant’s ability to resist and counteract biotic stressors. ABC transporters participate in the efflux of toxic compounds, such as anti-microbial secondary metabolites, from the plant cells. By doing so, they contribute to the detoxification processes that help plants combat pathogens and pests [78,79]. These transporters are also implicated in the transport of phytohormones, including auxin and abscisic acid (ABA), which play key roles in mediating plant responses to biotic stress [80]. Moreover, ABC transporters are known to be involved in the regulation of cuticle formation and composition, influencing the physical barrier that protects plants from pathogen invasion [81]. The transport of lipids, including those involved in cuticle formation, is a crucial function of ABC transporters in plant defense mechanisms. The expression of ABC transporter genes is often induced in response to pathogen attack, indicating their dynamic regulation in the face of biotic stress [82]. The upregulation of ABC transporters contributes to the active transport of defense-related molecules, facilitating an effective response against invading pathogens.

This study provided a comprehensive analysis of gene expression changes in coconut leaves under RPW infestation conditions. This study identified a significant number of DEGs under RPW infestation conditions. The highest number of DEGs was observed in certain comparison groups, particularly those involving RPW infestation under different population densities (e.g., B, C, and D densities) compared to control groups. This indicates a substantial transcriptional response of coconut leaves to RPW infestation, with both up-regulated and down-regulated genes being identified. The principal component analysis (PCA) and Pearson correlation analysis were performed to assess the reliability of the transcriptome data. Strong correlations between biological replicates were observed, indicating the consistency and reproducibility of the gene expression patterns across samples. Clear clustering of samples further affirmed the reliability of the data, supporting its suitability for subsequent analysis. Hierarchical clustering of DEGs revealed similar expression patterns across different sample groups. Genes showing similar expression changes under different experimental conditions may have related functions, suggesting common regulatory mechanisms in response to RPW infestation. Several genes were identified across different treatment comparisons and time points, providing insights into the molecular mechanisms underlying the progression of RPW infestation. Differential expression of specific genes, such as *E3 ubiquitin-protein ligase UNKL*, *glycerol-3-phosphate transporter*, *chitinase*, and *calmodulin*, indicates their potential roles in the response to RPW infestation. Expression patterns of these genes varied across different population densities and time points, highlighting the dynamic nature of the plant’s response to RPW infestation. KEGG pathway analysis revealed significant enrichment of DEGs in pathways related to metabolism, the organismal system, genetic information processing, and cellular processes. GO analysis identified functional groups associated with cellular processes, metabolic processes, response to stimulus, and binding activities. These analyses provide a comprehensive understanding of the biological processes and molecular functions involved in the response of coconut leaves to RPW infestation. Researchers performed a deep transcriptome analysis and identified more than 3300 genes linked to lipid fatty acids, tryptophan, and phenylpropanoid metabolism. Notably, essential genes were modulated in innate response through hormone crosstalk, such as auxin, jasmonate, and SA pathways. Moreover, a few WRKY transcription factors were also activated [83]. Genetic expression changes in coconut leaves following RPW attack have been investigated [84], where the researchers observed significant alterations in genetic expression patterns in coconut leaves post-RPW infestation, which highlighted the impact of this pest on the host plant’s molecular responses. Additionally, another study [85] conducted transcriptomic analyses, revealing dynamic changes in gene expression in coconut leaves upon RPW infestation. An investigation identified how coconut leaves respond physiologically and at the transcriptional level following attacks by RPW over various durations. Analysis of physiological data revealed that populations with higher RPW densities, particularly populations, exhibited elevated antioxidant enzyme activity. Among the 38,432 identified transcripts, 3984, 1981, 3925, and 2257 showed significant differential expression in comparisons of control and infected groups. These transcripts were notably associated with diverse pathways. Transcriptomic analysis revealed approximately 662 genes expressed consistently across various treatments are potential indicators for detecting RPW at various stages of infestation development, which totally complies with our current study [86]. Transcriptome profiles were assessed following 1 and 7 days of aphid infestation in soyabean and unveiled a susceptible reaction characterized by numerous transcript alterations, while only one transcript exhibited a change in the resistant response to aphids. Examination of phytohormone-related transcripts in the susceptible reaction revealed distinct hormone profiles at the two time points, indicating the ability of aphids to suppress hormone signals in susceptible plants [86]. Functional annotation identified 25 genes potentially linked to RPW reproduction, notably including Vg, alongside crucial genes like apolipophorin III, low-density lipoprotein receptor, and chorion protein in a study [87].

Despite these promising findings, certain anomalies were noted. In some groups and at specific stages of infestation, the changes in these diagnostic markers did not follow a consistent pattern. This suggests the presence of more complex processes at play during the infestation, which requires further exploration. Combining all the identified diagnostic markers could be a robust method for assessing infestation. This integrated approach could maximize the detection of infestation cases while minimizing the risk of misdiagnosis, providing a more accurate representation of the plant’s health status and the extent of the RPW attack. The findings of this study pave the way for more targeted interventions in managing RPW infestations, potentially leading to more effective and sustainable pest control methods in the future.

## 4. Materials and Methods

### 4.1. RPW Insects Attack Model

Coconut (*Cocos nucifera*), a Hainan tall cultivar, was grown at the National Coconut Germplasm Nursery of the Coconut Research Institute (Wenchang City, Hainan Province, China) [44]. Samples of leaves taken from healthy coconut plants were collected and utilized in subsequent experiments. Before raising the RPW, extensive monitoring of its population dynamics was conducted over several years in the fields of the Coconut Research Institute, Chinese Academy of Tropical Agricultural Science, Hainan. It was observed that RPW adults exhibit three peak periods annually: April to May, July to September, and November. It was also noted that RPW shows increased activity at 17:30 during these peak periods. The RPW specimens used in this study originated from a standardized insect source bred in a controlled climate chamber set at 28 ± 0.1 °C under long-day conditions (14 h of light and 10 h of darkness), with a relative humidity of 60 ± 0.5%. The construction of an RPW attack model was as follows: Four groups of RPW larvae were introduced to the triennial growth point leaves of coconut plants, initiating a natural infestation process. Each experimental set-up had three replicates. The composition of each group comprised 5 males and 7 females (group A), 10 males and 14 females (group B), 15 males and 21 females (group C), and 20 males and 28 females (group D), respectively, on 22 April 2023, at 17:30, after two days of adults mating and female oviposition. The leaf samples from the control (CK) and experimental treatments were collected in triplicate, immediately frozen in liquid nitrogen, and stored at −80 °C in a refrigerator until further processing. The extent of RPW damage inflicted upon individual coconut plants was categorized into three severity levels: light, moderate, and severe, with the standard of each level defined by the population density affecting a single plant. In accordance with the population density criteria outlined in the Chinese forestry industry standard (LY/T 1681—2006 Standard of Forest Pests Occurrence and Disaster) [88], distinct infection modes were established for varying RPW population densities. To ensure experimental integrity and minimize interference from extraneous biological factors, a single 3-year-old coconut tree was encased in gauze mesh and supported by a steel frame structure. This setup guaranteed that each cohort of healthy coconut trees, subjected to different RPW density inoculations, underwent treatment in isolation. There was also a control group (CK) without any insect infestation. Infected leaf samples were collected, and the gas chromatography–mass spectrometry (GC–MS)-based metabolomic method was employed to identify chemical changes in RPW-infected coconut plant leaves at different infection times and attack densities. Respiration indicators such as CO_2_ concentration, stomatal conductance, water vapor pressure deficiency, net photosynthetic rate, evapotranspiration rate, and water use efficiency were measured according to the described protocol [89]. Leaves of coconut plants exceeding dimensions of approximately 2 cm × 2 cm were utilized in this study. The concentration of CO_2_ was assessed using a portable gas analyzer (LI-COR, Lincoln, NE, USA) positioned in proximity to the leaves. Measurements of CO_2_ concentration were taken from at least three leaves, and readings were recorded once a stable measurement was achieved (typically within 30–60 s). The evapotranspiration rate was calculated based on the mole fraction of water vapor at the outlet of the leaf chamber (mol mol^−1^) and the mole fraction of water vapor at the inlet of the leaf chamber (mol mol^−1^). Water use efficiency (WUE) was determined as either the ratio of net photosynthetic rate to transpiration rate or the ratio of biomass produced to water consumed. This metric reflects the effectiveness of plants in utilizing water in relation to their growth and photosynthetic processes. The net photosynthetic rate was quantified by assessing the exchange of CO_2_ and water vapor between the leaf and the atmosphere. Stomatal conductance was computed using the mole fraction of water vapor at saturation, considering that the leaf is saturated with water vapor at its actual temperature, the transpiration rate (mol m^−2^ s^−1^), and the mole fraction of water vapor at the outlet of the leaf chamber (mol mol^−1^). Additionally, chlorophyll (SPAD) levels at 10, 20, 30, 40, 50, 70, and 100 DAI from four different orientations (east, south, west, and north) of infected leaves were also assessed [90,91]. Only chlorophyll levels were analyzed at 10, 20, 30, 40, 50, 70, and 100 DAI, while other parameters were accessed at 10 DAI, 20 DAI, 30 DAI, 40 DAI, and 50 DAI. In the case of transcriptome analysis, the samples were collected and named according to the following conditions: 2023. 05.03 (10 DAI), 2023. 05.13 (20 DAI), 2023. 05.23 (30 DAI), 2023. 06.02 (40 DAI), and 2023. 06.12 (50 DAI).

### 4.2. Sample Preparation 

Approximately 50 mg (±5%) of *C. nucifera* leaves were transferred into a 2 mL centrifuge tube. The leaf samples from both the control and experimental treatments were collected in triplicate. Samples were collected from three biological repeats from each experimental condition. A mixed solution of 0.5 mL of acetonitrile/isopropanol/water (3:3:2, *v*/*v*/*v*) that were stored at −20 ℃ was added. Subsequently, about 3–4 zirconium beads (2 mm) were introduced and subjected to 30 Hz shock for 20 s using a high-flux tissue grinder (allowed to stand for 10 s, repeated for eight cycles, followed by an ice water bath for 5 min). Then, 0.5 mL of CH3CN:C3H8O:H_2_O (3:3:2, *v*/*v*/*v*) was added, and the mixture was sonicated for 5 min in an ice water bath, followed by centrifugation for 2 min at 12,000 rpm (4 °C). Afterward, 500 μL of the supernatant solution was concentrated to dryness (for approximately 8–10 h). The residue was reconstituted with 80 μL of a 20 mg/mL methoxyamine solution. Finally, 100 μL of N, O-Bis (trimethylsilyl) trifluoroacetamide with trimethylchlorosilane (99:1) reagent was added, and the mixture was incubated at 70 ℃ for 90 min. After the final centrifugation at 12,000 rpm for 1 min, 90–100 μL of the supernatant was collected and utilized for GC-MS analysis within 24 h.

### 4.3. GC-MS Analysis 

For this analysis, A column (Model: J&W DB-5MS (5% Phenyl Methyl Siloxane), Agilent Technologies, Inc., Santa Clara, CA, USA) was used in conjunction with a GC System (Model: 7890A, Agilent Technologies, Inc., Santa Clara, CA, USA) and an inert MSD with a Triple-Axis Detector (Model: 5975C, Agilent Technologies, Inc., Santa Clara, CA, USA). The injector temperature was maintained at 250 °C, while the GC-MS ran in electron ionization mode at 70 eV. The carrier gas was helium, flowing at a rate of 1 milliliter per minute. The sample was injected in split-less mode using a 1 µL aliquot. After two minutes at 50 °C, the oven’s temperature was raised by 10 °C every minute for five minutes, reaching 280 °C. The mass range of the mass selective detector in scan mode was *m*/*z* 50–550 amu. The ion source and quadrupole temperatures were maintained at 230 °C and 150 °C, respectively. Compound identification relied on the comparison of retention times and mass spectra. The analysis involved three sample replicates to ensure the reliability of the experimental results.

### 4.4. Metabolites Analysis

Metabolite analysis commenced with data preprocessing to derive *m*/*z* values, retention time (RT), and peak intensity. The identification of metabolites involved a comprehensive comparison of *m*/*z* values, RT, and fragmentation patterns with established standards from the Human Metabolome Database (HMDB; http://www.hmdb.ca/) (accessed on 20 November 2023). The Variable Importance of Projection (VIP) score from the orthogonal projections to latent structures–discriminant analysis model was employed to identify overarching metabolic alterations within comparable groups. Metabolites meeting the criteria of a *t*-test-adjusted *p*-value (*p*-adj) ≤ 0.05 and VIP ≥ 1 were classified as differential metabolites. 

### 4.5. Statistical Analysis 

The statistical analysis of CO_2_ concentration, stomatal conductance, water vapor pressure deficiency, net photo-synthetic rate, evapotranspiration rate, water use efficiency, and chlorophyl levels was analyzed using a two-way analysis of variance (ANOVA), followed by Duncan’s Multiple Range Test (DMRT) for post hoc mean comparison at a significance level of *p* ≤ 0.05, conducted using GenStat, 12th edition (VSN International Ltd., Hemel Hempstead, UK).The multivariate statistical analysis was executed using the Bioconductor R package on the Majorbio Cloud Platform (https://cloud.majorbio.com) (accessed on 12 November 2023). A principal component analysis (PCA) was carried out using an unsupervised method.

### 4.6. RNA Extraction and Quality Control

The experimental process of transcriptome sequencing included RNA extraction, library construction, RNA sequencing, and functional annotation of identified differentially expressed genes. Total RNA was extracted from control and infested coconut leaves. RNA was extracted from 3 g of leaf samples using TRIzol^®^ Reagent (Invitrogen, Carlsbad, CA, USA), according to the manufacturer’s instructions [92]. Samples were first homogenized using TRIZOL Reagent. The homogenized samples were then incubated at 15 to 30 °C for 5 min to dissociate nucleoprotein complexes. Subsequently, 0.2 mL of chloroform was added per 1 mL of TRIZOL Reagent. The tubes were capped, shaken vigorously, and incubated at 15 to 30 °C for 2 to 3 min, followed by centrifugation at 12,000× *g* for 15 min at 2 to 8 °C to separate into aqueous and organic phases. RNA remains in the aqueous phase. RNA precipitation was performed using 0.5 mL of isopropyl alcohol per 1 mL of TRIZOL Reagent. After incubation at 15 to 30 °C for 10 min and centrifugation at 12,000× *g* for 10 min at 2 to 8 °C, RNA appears as a gel-like pellet. The RNA pellet was then partially dissolved to avoid complete drying, maintaining its solubility for downstream applications. After that, the extracted RNA was treated with DNase I (Invitrogen). RNA integrity and any DNA contamination were analyzed through agarose gel electrophoresis. RNA concentration was measured using a fluorometer (Model: Qubit 2.0, Thermo Fisher Scientific, Waltham, MA, USA). The accurate RNA integrity was detected using a Bioanalyzer (Model: 2100, Agilent Technologies, Inc., Santa Clara, CA, USA). 

### 4.7. Library Construction and RNA-Sequencing

mRNA samples were obtained by removing ribosomal RNA from the total RNA. Then, a fragmentation buffer was added to break the RNA into short fragments. This fragmentation buffer contains a Zn^2+^-containing damping fluid with the termination reaction solution (200 mM EDTA, pH 8.0). Using the short fragment of RNA as a template, six-base random primers (random hexamers) were used to synthesize the first-strand cDNA. Hexamers were chosen by computationally excluding hexamers that are complementary to rRNA from a library of random hexamer primers [93]. and then the buffer and dNTPs were added, along with DNA polymerase I to synthesize double-stranded cDNA, and then AMPure XP beads to purify the double-stranded cDNA. The purified double-stranded cDNA was then end-repaired, A-tailed, and connected to sequencing adapters. Then, AMPure XP beads were used for fragment size selection, and finally, PCR enrichment was performed to obtain the final cDNA library. After the library construction is completed, the quality of the library is tested using Qubit 2.0 and Agilent 2100 to detect preliminary quantification and insert size of the library, respectively. After the library inspection was qualified, different libraries were pooled according to the target off-machine data volume and sequenced using the Illumina platform (San Diego, CA, USA). Raw data are usually provided in fastq format and mainly contains sequence information of sequencing fragments and corresponding sequencing quality information. Fastp (Version 1.17.1) [94] was used to perform strict quality control. 

### 4.8. Identification and Functional Annotation of Differentially Expressed Genes

Based on the FPKM (fragments per kilobase of transcript per million fragments mapped) values, significant differences in transcript abundance were identified in at least one pairwise comparison. DESeq2 [95] was used to carry out the differential expression analysis, and a Q value ≤ 0.05 was used. Genes that had a Q value of less than 0.05 and a fold change of greater than or equal to 2.0 were deemed substantially differentially expressed. The Kyoto Encyclopedia of Genes and Genomes (KEGG) [96] and Gene Ontology (GO) [97] databases were used to undertake functional annotation and enrichment analysis of the DEGs. For every KEGG pathway and GO term, the quantity of DEGs and the significant enrichment outcomes were determined.

## 5. Conclusions

Most of the world’s date-producing regions, particularly those in the Middle East and North Africa, are affected by RPW. Currently, controlling the weevil involves the application of insecticides, the elimination of infected palm trees, and mass-trapping of adult weevils. However, there is an issue with the overuse of pesticide treatments. This could potentially be addressed by developing more effective early detection approaches, which, when combined with innovative control strategies, would result in more long-lasting control. This study provides insights into the complex interplay between coconut plant physiology and RPW infestation. The findings of the current study indicate that RPW infestation triggered a decrease in photosynthetic activity at the late stage of infection. At the same time, the chlorophyll levels of the east and west side leaves were reduced at both the early and late stages of infestation. Several metabolites are also identified that could be used as potential markers to identify different stages of infestation. The impact on metabolic pathways, including glutathione, galactose, and glycerolipid metabolism, suggests potential avenues for early detection and targeted pest management in coconut plants. The findings of this study are consistent with previous research demonstrating the impact of RPW infestation on the molecular responses of coconut leaves. Transcriptomic analyses have been conducted in other plant species infested with insect pests, highlighting the importance of understanding the molecular mechanisms underlying plant–insect interactions. The current study provided valuable insights into the molecular responses of coconut leaves to RPW infestation, shedding light on key genes, pathways, and biological processes involved in the plant’s defense mechanisms. This knowledge could inform the development of effective strategies for RPW management and crop protection. 

## Data Availability

All the data and resources generated for this study are included in the article.

## Supplementary Materials

The following supporting information can be downloaded at: https://www.mdpi.com/article/10.3390/plants13141928/s1, Table S1: Summary of results for various metabolic pathways, including total gene counts, the number of hits, raw *p*-values, −log(*p*) values, Holm–Bonferroni method adjusted *p*-values, False Discovery Rate (FDR), and impact scores of Group A vs. CK (No insects); Table S2: Summary of results for various metabolic pathways, including total gene counts, the number of hits, raw *p*-values, −log(*p*) values, Holm–Bonferroni method adjusted *p*-values, False Discovery Rate (FDR), and impact scores of Group B vs. CK (No insects); Table S3: Summary of results for various metabolic pathways, including total gene counts, the number of hits, raw *p*-values, −log(*p*) values, Holm–Bonferroni method adjusted *p*-values, False Discovery Rate (FDR), and impact scores of Group C vs. CK (No insects); Table S4: Summary of results for various metabolic pathways, including total gene counts, the number of hits, raw *p*-values, −log(*p*) values, Holm–Bonferroni method adjusted *p*-values, False Discovery Rate (FDR), and impact scores of Group D vs. CK (No insects); Table S5: Total number of differentially expressed genes; number of Down-regulated and Up-regulated genes.
