# Supplementary material for: Molecular Insights into Red Palm Weevil Resistance Mechanisms of Coconut (Cocos nucifera) Leaves
Source: Plants (Basel). 2024 Jul 12;13(14):1928. doi: 10.3390/plants13141928 (PMC11280253; doi:10.3390/plants13141928)
Supplement: Supplementary file 1 [file plants-13-01928-s001.zip › plants-3041724-supplementary.pdf]

Supplementary Table S1. Summary of results for various metabolic pathways, including total gene counts, the number of hits, raw p-values, -log(p) values, Holm–Bonferroni method adjusted p-values, False Discovery Rate (FDR), and impact scores of Group A vs CK (No insects).

	Total	Hits	Raw p	-log(p)	Holm adjust	FDR	Impact
**Glutathione metabolism**	38	4	0.001453	6.5344	0.15253	0.071434	0.16013
**ABC transporters Processing**	138	7	0.002022	6.2038	0.21026	0.071434	0.050725
**Ascorbate and aldarate metabolism**	50	4	0.004033	5.5133	0.41537	0.087322	0.13559
**Sphingolipid metabolism**	25	3	0.004119	5.4922	0.42013	0.087322	0.20755
**Phenylalanine, tyrosine and tryptophan biosynthesis**	34	3	0.009857	4.6196	0.99554	0.17414	0.072581
**Glycerolipid metabolism**	38	3	0.01339	4.3132	1	0.20277	0.10582
**Butanoate metabolism**	42	3	0.01756	4.0421	1	0.23268	0.090909
**Citrate cycle (TCA cycle)**	20	2	0.028174	3.5694	1	0.32928	0.077778
**Fructose and mannose metabolism**	54	3	0.033973	3.3822	1	0.32928	0.030303
**Nicotinate and nicotinamide metabolism**	55	3	0.035605	3.3353	1	0.32928	0.06087
**Pentose and glucuronate interconversions**	56	3	0.037277	3.2894	1	0.32928	0.039216
**Fatty acid biosynthesis**	58	3	0.04074	3.2005	1	0.33219	0.023952
**Glyoxylate and dicarboxylate metabolism**	62	3	0.04814	3.0336	1	0.34687	0.26996
**Cutin, suberine and wax biosynthesis**	27	2	0.049086	3.0142	1	0.34687	0.054545
**Alanine, aspartate and glutamate metabolism**	28	2	0.052422	2.9484	1	0.3473	0.10377
**Thiamine metabolism**	31	2	0.062898	2.7662	1	0.3704	0.042553
**Glycolysis / Gluconeogenesis**	31	2	0.062898	2.7662	1	0.3704	0.056426
**C5-Branched dibasic acid metabolism**	34	2	0.074018	2.6034	1	0.41294	0.047619
**Starch and sucrose metabolism**	37	2	0.085718	2.4567	1	0.4543	0.35028
**Inositol phosphate metabolism**	47	2	0.12815	2.0545	1	0.62589	0.044164
**Photosynthesis**	11	1	0.13689	1.9886	1	0.62589	0.076923
**Lysine degradation**	50	2	0.14171	1.954	1	0.62589	0.05102
**Glycine, serine and threonine metabolism**	50	2	0.14171	1.954	1	0.62589	0.13415
**Phosphonate and phosphinate metabolism**	56	2	0.16967	1.7739	1	0.69972	0.030303

**Amino sugar and nucleotide sugar metabolism**	108	3	0.17163	1.7624	1	0.69972	0.066667
**Oxidative phosphorylation**	16	1	0.19287	1.6457	1	0.75719	0.05
**Phenylpropanoid biosynthesis**	68	2	0.22788	1.4789	1	0.85221	0.024038
**Caffeine metabolism**	22	1	0.25537	1.3651	1	0.85221	0.03125
**Taurine and hypotaurine metabolism**	22	1	0.25537	1.3651	1	0.85221	0.035714
**Biosynthesis of unsaturated fatty acids**	74	2	0.25759	1.3564	1	0.85221	0.041667
**Carbon fixation in photosynthetic organisms**	23	1	0.26531	1.3269	1	0.85221	0.021459
**Arginine biosynthesis**	23	1	0.26531	1.3269	1	0.85221	0.047761
**Arginine and proline metabolism**	78	2	0.27745	1.2821	1	0.865	0.013158
**Biosynthesis of various secondary metabolites - part 2**	81	2	0.29235	1.2298	1	0.88539	0.021505
**Phosphatidylinositol signaling system**	29	1	0.3223	1.1323	1	0.94901	0.038229
**Pyruvate metabolism**	31	1	0.34032	1.0779	1	0.97395	0.019868
**beta-Alanine metabolism**	32	1	0.34915	1.0522	1	0.97395	0.033333
**Purine metabolism**	95	2	0.36114	1.0185	1	0.98155	0.007129
**Lysine biosynthesis**	35	1	0.37496	0.98094	1	0.99364	0.066667
**Fatty acid elongation**	40	1	0.41576	0.87765	1	1	0.016949
**Cyanoamino acid metabolism**	45	1	0.45395	0.78978	1	1	0.019417
**Histidine metabolism**	47	1	0.46853	0.75816	1	1	0.030303
**Fatty acid degradation**	50	1	0.48969	0.71399	1	1	0.009375
**Aminoacyl-tRNA biosynthesis**	52	1	0.50333	0.68651	1	1	0.018182
**Glycerophospholipid metabolism**	52	1	0.50333	0.68651	1	1	0.059271
**Steroid biosynthesis**	58	1	0.54216	0.6122	1	1	0.021127
**Tropane, piperidine and pyridine alkaloid biosynthesis**	68	1	0.60036	0.51023	1	1	0.013158
**Tyrosine metabolism**	78	1	0.6513	0.42879	1	1	0.006494
**Sesquiterpenoid and triterpenoid biosynthesis**	88	1	0.69585	0.36261	1	1	0.03
**Porphyrin and chlorophyll metabolism**	142	1	0.85561	0.15595	1	1	0.001873
**Galactose metabolism**	46	9	4.53E-09	19.212	4.80E-07	4.80E-07	0.29091

Supplementary Table S2. Summary of results for various metabolic pathways, including total gene counts, the number of hits, raw p-values, -log(p) values, Holm–Bonferroni method adjusted p-values, False Discovery Rate (FDR), and impact scores of Group B vs CK (No insects)

	Total	Hits	Raw p	-log$_{(p)}$	Holm adjust	FDR	Impact
**Galactose metabolism**	46	3	0.006979	4.9648	0.7398	0.28034	0.10909
**Inositol phosphate metabolism**	47	3	0.007412	4.9047	0.77821	0.28034	0.050473
**Ascorbate and aldarate metabolism**	50	3	0.008803	4.7327	0.91547	0.28034	0.11864
**Citrate cycle (TCA cycle)**	20	2	0.012554	4.3777	1	0.28034	0.033333
**Fatty acid biosynthesis**	58	3	0.013224	4.3257	1	0.28034	0.023952
**Sphingolipid metabolism**	25	2	0.019295	3.9479	1	0.29593	0.16981
**Phenylpropanoid biosynthesis**	68	3	0.020259	3.8992	1	0.29593	0.033654
**Cutin, suberine and wax biosynthesis**	27	2	0.022334	3.8016	1	0.29593	0.054545
**ABC transporters**	138	4	0.029858	3.5113	1	0.33892	0.028986
**Biosynthesis of various secondary metabolites - part 2**	81	3	0.031974	3.4428	1	0.33892	0.032258
**Butanoate metabolism**	42	2	0.050562	2.9846	1	0.48723	0.068182
**Nicotinate and nicotinamide metabolism**	55	2	0.08139	2.5085	1	0.68459	0.017391
**Pentose and glucuronate interconversions**	56	2	0.083959	2.4774	1	0.68459	0.058824
**Biosynthesis of unsaturated fatty acids**	74	2	0.13395	2.0103	1	0.96627	0.041667
**Tyrosine metabolism**	78	2	0.14585	1.9252	1	0.96627	0.012987
**Arginine and proline metabolism**	78	2	0.14585	1.9252	1	0.96627	0.15461
**Carbon fixation in photosynthetic organisms**	23	1	0.18199	1.7038	1	1	0.021459
**Alanine, aspartate and glutamate metabolism**	28	1	0.21707	1.5275	1	1	0.025157
**Phosphatidylinositol signaling system**	29	1	0.22391	1.4965	1	1	0.038229
**Pyruvate metabolism**	31	1	0.23742	1.4379	1	1	0.019868
**Glycolysis / Gluconeogenesis**	31	1	0.23742	1.4379	1	1	0.040752

**Amino sugar and nucleotide sugar metabolism**	108	2	0.2403	1.4259	1	1	0.012121
**beta-Alanine metabolism**	32	1	0.24408	1.4102	1	1	0.033333
**C5-Branched dibasic acid metabolism**	34	1	0.25725	1.3577	1	1	0.02381
**Phenylalanine, tyrosine and tryptophan biosynthesis**	34	1	0.25725	1.3577	1	1	0.040323
**Starch and sucrose metabolism**	37	1	0.27658	1.2852	1	1	0.15254
**Glycerolipid metabolism**	38	1	0.28292	1.2626	1	1	0.002646
**Glutathione metabolism**	38	1	0.28292	1.2626	1	1	0.01634
**Fatty acid elongation**	40	1	0.29543	1.2193	1	1	0.016949
**Fatty acid degradation**	50	1	0.35487	1.036	1	1	0.009375
**Fructose and mannose metabolism**	54	1	0.37726	0.97481	1	1	0.007576
**Phosphonate and phosphinate metabolism**	56	1	0.38817	0.9463	1	1	0.015152
**Steroid biosynthesis**	58	1	0.3989	0.91904	1	1	0.021127
**Glyoxylate and dicarboxylate metabolism**	62	1	0.41981	0.86795	1	1	0.020913
**Tropane, piperidine and pyridine alkaloid biosynthesis**	68	1	0.44986	0.79883	1	1	0.013158
**Flavonoid biosynthesis**	74	1	0.47839	0.73732	1	1	0.015625
**Sesquiterpenoid and triterpenoid biosynthesis**	88	1	0.53952	0.61708	1	1	0.03
**Ubiquinone and other terpenoid-quinone biosynthesis**	92	1	0.55567	0.58759	1	1	0.006135
**Isoquinoline alkaloid biosynthesis**	122	1	0.66047	0.4148	1	1	0.006579

Supplementary Table S3. Summary of results for various metabolic pathways, including total gene counts, the number of hits, raw p-values, -log(p) values, Holm–Bonferroni method adjusted p-values, False Discovery Rate (FDR), and impact scores of Group C vs CK (No insects)

	Total	Hits	Raw p	-log(p)	Holm adjust	FDR	Impact
**Glycerolipid metabolism**	38	3	0.002762	5.8917	0.2845	0.073197	0.16402
**Starch and sucrose metabolism**	37	1	0.24659	1.4	1	1	0.15254
**Pentose and glucuronate interconversions**	56	4	0.000742	7.2056	0.077956	0.039349	0.11765
**Sphingolipid metabolism**	25	1	0.17387	1.7495	1	1	0.09434
**Glycerophospholipid metabolism**	52	1	0.32884	1.1122	1	1	0.080547
**Ascorbate and aldarate metabolism**	50	1	0.3184	1.1445	1	1	0.067797
**Stilbenoid, diarylheptanoid and gingerol biosynthesis**	25	1	0.17387	1.7495	1	1	0.051282
**Glycolysis / Gluconeogenesis**	31	3	0.001525	6.4858	0.1586	0.053884	0.047022
**Galactose metabolism**	46	4	0.000348	7.9646	0.036839	0.036839	0.045455
**Phenylalanine, tyrosine and tryptophan biosynthesis**	34	1	0.22901	1.474	1	1	0.040323
**Glutathione metabolism**	38	1	0.25236	1.3769	1	1	0.039216
**Citrate cycle (TCA cycle)**	20	2	0.009692	4.6365	0.97889	0.17122	0.033333
**Sesquiterpenoid and triterpenoid biosynthesis**	88	1	0.4924	0.70835	1	1	0.03
**ABC transporters**	138	4	0.019064	3.96	1	0.28868	0.028986
**Inositol phosphate metabolism**	47	3	0.005072	5.2839	0.51739	0.10754	0.025237
**Ubiquinone and other terpenoid-quinone biosynthesis**	92	1	0.50806	0.67716	1	1	0.02454
**Biosynthesis of various secondary metabolites - part 2**	81	2	0.12472	2.0817	1	1	0.021505
**Carbon fixation in photosynthetic organisms**	23	1	0.16111	1.8257	1	1	0.021459
**Steroid biosynthesis**	58	1	0.35926	1.0237	1	1	0.021127
**Pyruvate metabolism**	31	1	0.21103	1.5557	1	1	0.019868
**Flavonoid biosynthesis**	74	1	0.43401	0.83469	1	1	0.015625
**Amino sugar and nucleotide sugar metabolism**	108	3	0.046961	3.0584	1	0.62223	0.015152

**Phosphonate and phosphinate metabolism**	56	1	0.34927	1.0519	1	1	0.015152
**Phosphatidylinositol signaling system**	29	1	0.19883	1.6153	1	1	0.012072
**Phenylpropanoid biosynthesis**	68	1	0.40702	0.8989	1	1	0.009615
**Folate biosynthesis**	58	1	0.35926	1.0237	1	1	0.005814
**Glyoxylate and dicarboxylate metabolism**	62	1	0.37879	0.97077	1	1	0.005703
**Alanine, aspartate and glutamate metabolism**	28	1	0.19266	1.6469	1	1	0.003145

Supplementary Table S4. Summary of results for various metabolic pathways, including total gene counts, the number of hits, raw p-values, -log(p) values, Holm–Bonferroni method adjusted p-values, False Discovery Rate (FDR), and impact scores of Group D vs CK (No insects)

	**Total**	**Hits**	**Raw p**	**-log(p)**	**Holm adjust**	**FDR**	**Impact**
**ABC transporters**	138	7	0.000581	7.4506	0.061014	0.030798	0.050725
**Pentose and glucuronate interconversions**	56	4	0.002895	5.8447	0.3011	0.1023	0.098039
**Starch and sucrose metabolism**	37	3	0.0071	4.9476	0.73134	0.16221	0.22034
**Glycerolipid metabolism**	38	3	0.007652	4.8728	0.78047	0.16221	0.11376
**Citrate cycle (TCA cycle)**	20	2	0.019237	3.9509	1	0.33986	0.077778
**Fatty acid biosynthesis**	58	3	0.024115	3.7249	1	0.36517	0.024701
**Sphingolipid metabolism**	25	2	0.02936	3.5281	1	0.38902	0.11321
**Glycolysis / Gluconeogenesis**	31	2	0.043697	3.1305	1	0.47708	0.043887
**Biosynthesis of unsaturated fatty acids**	74	3	0.045008	3.1009	1	0.47708	0.0625
**Phenylalanine, tyrosine and tryptophan biosynthesis**	34	2	0.051661	2.963	1	0.49783	0.064516
**Butanoate metabolism**	42	2	0.075137	2.5884	1	0.66371	0.034091
**Inositol phosphate metabolism**	47	2	0.091234	2.3943	1	0.74391	0.012618
**Ascorbate and aldarate metabolism**	50	2	0.10134	2.2893	1	0.76729	0.084746
**Photosynthesis**	11	1	0.1131	2.1795	1	0.78728	0.076923
**Nicotinate and nicotinamide metabolism**	55	2	0.11883	2.13	1	0.78728	0.052174
**Glyoxylate and dicarboxylate metabolism**	62	2	0.14448	1.9346	1	0.90086	0.072243
**Oxidative phosphorylation**	16	1	0.16029	1.8308	1	0.94393	0.05
**Taurine and hypotaurine metabolism**	22	1	0.21369	1.5432	1	1	0.035714

**Biosynthesis of various secondary metabolites - part 2**	81	2	0.2186	1.5205	1	1	0.021505
**Carbon fixation in photosynthetic organisms**	23	1	0.22227	1.5039	1	1	0.021459
**Arginine biosynthesis**	23	1	0.22227	1.5039	1	1	0.047761
**Cutin, suberine and wax biosynthesis**	27	1	0.25565	1.3639	1	1	0.018182
**Alanine, aspartate and glutamate metabolism**	28	1	0.26378	1.3326	1	1	0.078616
**Pyruvate metabolism**	31	1	0.28764	1.246	1	1	0.019868
**C5-Branched dibasic acid metabolism**	34	1	0.31075	1.1688	1	1	0.02381
**Pentose phosphate pathway**	35	1	0.31829	1.1448	1	1	0.00578
**Lysine biosynthesis**	35	1	0.31829	1.1448	1	1	0.066667
**Amino sugar and nucleotide sugar metabolism**	108	2	0.32784	1.1152	1	1	0.030303
**Glutathione metabolism**	38	1	0.34043	1.0776	1	1	0.039216
**Fatty acid elongation**	40	1	0.35479	1.0362	1	1	0.016949
**alpha-Linolenic acid metabolism**	44	1	0.38262	0.96072	1	1	0.14964
**Histidine metabolism**	47	1	0.40272	0.90952	1	1	0.030303
**Fatty acid degradation**	50	1	0.42218	0.86233	1	1	0.009375
**Glycine, serine and threonine metabolism**	50	1	0.42218	0.86233	1	1	0.018293
**Lysine degradation**	50	1	0.42218	0.86233	1	1	0.040816
**Glycerophospholipid metabolism**	52	1	0.4348	0.83286	1	1	0.00152
**Fructose and mannose metabolism**	54	1	0.44716	0.80483	1	1	0.10606
**Phosphonate and phosphinate metabolism**	56	1	0.45926	0.77814	1	1	0.015152
**Steroid biosynthesis**	58	1	0.47109	0.7527	1	1	0.021127
**Phenylpropanoid biosynthesis**	68	1	0.52659	0.64133	1	1	0.004808
**Tropane, piperidine and pyridine alkaloid biosynthesis**	68	1	0.52659	0.64133	1	1	0.013158
**Flavonoid biosynthesis**	74	1	0.55712	0.58498	1	1	0.007813
**Tyrosine metabolism**	78	1	0.5764	0.55096	1	1	0.006494
**Sesquiterpenoid and triterpenoid biosynthesis**	88	1	0.62108	0.4763	1	1	0.03
**Galactose metabolism**	46	7	3.98E-07	14.736	4.22E-05	4.22E-05	0.18182

Supplementary Table S5. Total number of differentially expressed genes; number of Down-regulated and Up-regulated genes.

Groups	Total DEGs	Down-regulated	Up-regulated
0428A_vs_0428B	1020	548	472
0428A_vs_0428C	2413	1180	1233
0428A_vs_0428D	2912	1479	1433
0428A_vs_0503A	4347	2335	2012
0428A_vs_0508A	2219	779	1440
0428A_vs_0513A	504	304	200
0428A_vs_0518A	3303	1764	1539
0428B_vs_0428C	2094	1034	1060
0428B_vs_0428D	2538	1286	1252
0428B_vs_0503B	5070	2737	2333
0428B_vs_0508B	603	310	293
0428B_vs_0513B	353	230	123
0428B_vs_0518B	3687	1745	1942
0428C_vs_0428D	1809	979	830
0428C_vs_0503C	5346	2850	2496
0428C_vs_0508C	2407	884	1523
0428C_vs_0513C	932	365	567
0428C_vs_0518C	3483	1703	1780
0428CK_vs_0428A	3809	1761	2048
0428CK_vs_0428B	3806	1824	1982
0428CK_vs_0428C	3984	1827	2157
0428CK_vs_0428D	1981	792	1189
0428CK_vs_0503CK	5347	2718	2629
0428CK_vs_0508CK	2547	1201	1346
0428CK_vs_0513CK	1754	783	971
0428CK_vs_0518CK	5057	2322	2735
0428D_vs_0503D	4073	2174	1899
0428D_vs_0508D	2200	845	1355
0428D_vs_0513D	1505	639	866
0428D_vs_0518D	3106	1716	1390
0503A_vs_0503B	1451	838	613
0503A_vs_0503C	2718	1154	1564

0503A_vs_0503D	2556	1302	1254
0503A_vs_0508A	2745	1103	1642
0503A_vs_0513A	3388	1741	1647
0503A_vs_0518A	638	401	237
0503B_vs_0503C	3293	1497	1796
0503B_vs_0503D	2380	1139	1241
0503B_vs_0508B	3041	1389	1652
0503B_vs_0513B	4277	2160	2117
0503B_vs_0518B	1138	356	782
0503C_vs_0503D	3719	2055	1664
0503C_vs_0508C	3312	1423	1889
0503C_vs_0513C	3708	1706	2002
0503C_vs_0518C	941	375	566
0503CK_vs_0503A	3707	1796	1911
0503CK_vs_0503B	3097	1491	1606
0503CK_vs_0503C	5373	2632	2741
0503CK_vs_0503D	1958	816	1142
0503CK_vs_0508CK	3357	1638	1719
